# Evaluation of lifespan promoting effects of biofortified wheat in *Drosophila melanogaster*

**DOI:** 10.1016/j.exger.2022.111697

**Published:** 2022-01-10

**Authors:** Manish Pandey, Sakshi Bansal, Geetanjali Chawla

**Affiliations:** RNA Biology Laboratory, Regional Centre for Biotechnology, NCR Biotech Science Cluster, Faridabad 121001, Haryana, India

**Keywords:** Lifespan, Biofortified, Dietary restriction, Ageing, High fat diet, Antioxidant, Anthocyanin

## Abstract

Evaluation of nutritionally enhanced biofortified dietary interventions that increase lifespan may uncover cost-effective and sustainable approaches for treatment of age-related morbidities and increasing healthy life expectancy. In this study, we report that anthocyanin rich, high yielding crossbred blue wheat prolongs lifespan of *Drosophila melanogaster* in different dietary contexts. In addition to functioning as an antioxidant rich intervention, the biofortified blue wheat also works through modulating expression of DR pathway genes including *AMPK alpha*, *SREBP*, *PEPCK* and *Cry*. Supplementation with blue- or purple-colored wheat provided better protection against paraquat-induced oxidative stress than control diet and increased survivability of flies in which *superoxide dismutase* 2 was knocked down conditionally in adults. Lastly, our findings indicate that supplementing biofortified blue wheat formulated diet prevented the decrease in lifespan and cardiac structural pathologies associated with intake of high fat diet. Overall, our findings indicate that plant-based diets formulated with biofortified cereal crops promote healthy ageing and delay progression of diseases that are exacerbated by accumulation of oxidative damage.

## 1 Introduction

Advances in ageing research in *Drosophila melanogaster* and other model organisms indicate that dietary manipulations based on nutrient restriction and few antioxidants promote healthy ageing (Gonzalez-Freire et al., 2020; Luersen et al., 2019; Magwere et al., 2006; Minor et al., 2010; Ng et al., 2014). The free radical theory of ageing is one of the classical theories of ageing and proposes that ageing results from accumulation of oxidative damage to cells and tissues of the body as a result of aerobic metabolism (Harman, 1956). Hence, a major focus of research has been to test the potential of antioxidant compounds to delay ageing and prevent age-related diseases and disabilities. However, the evidence that supports this theory has only been able to correlate ageing with oxidative damage and many of the experiments involving manipulations have produced variable effects (Blackett and Hall, 1981; Morley and Trainor, 2001). Clinical studies with single antioxidant supplements such as beta carotene, vitamin A, Vitamin C and Vitamin E have demonstrated that single antioxidants do not protect against chronic diseases including heart disease and cancer (Bjelakovic et al., 2007; Grodstein et al., 2007; Lee et al., 2005). One plausible reason for these results might be that antioxidants work best in combination with other micronutrients, other antioxidants or phytochemicals and supplementation with a single antioxidant may not produce the same effect. Differences in the amounts and chemical form of supplements and natural foods might also influence the effects observed in clinical trials. In contrast to the above-described studies, there is substantial evidence to suggest that intake of antioxidant-rich fruits, vegetables and whole grains consumed in their natural context is associated with a lower risk for chronic oxidative stress-related diseases like cardiovascular diseases (Aune et al., 2017; Bhupathiraju et al., 2013; Cao et al., 2020; Joshipura et al., 2001; Kang et al., 2005; Miller et al., 2017; Szabo et al., 2021). Whether the protection mediated by plant-based diets is due to the antioxidants and/or other substances in the diet is unclear (Carlsen et al., 2010). Thus, assessment of the natural whole foods' rich in networks of antioxidants and the helper substances is needed to gain an under-standing of the ageing pathways that are modulated by these interventions. Anthocyanins are one class of natural dietary phytochemicals that function as antioxidants and are responsible for the black, blue, purple, red and orange colors of many fruits and vegetables (Bors et al., 1990; Stintzing et al., 2002; Wang et al., 1997). Thus, evaluation of plant-based diets and an understanding of the mechanisms underlying the beneficial effects can provide an acceptable solution for enhancing healthy lifespan and delaying age-related diseases.

Enhancement of nutrient quality and bioavailability by bio-fortification of food crops has been employed as a strategy by developing countries to increase the availability of essential nutrients and other health promoting compounds such as anthocyanins (Bouis and Saltzman, 2017; Garg et al., 2018; Khush et al., 2012). In addition to serving as a substitute for dietary supplements, augmentation of nutrient content is a long-term solution for counteracting malnutrition and promoting health in humans. However, careful evaluation in animal models is critical to gain an understanding of the long-term impact of consuming biofortified foods. Given the mechanistic conservation of antioxidative mechanisms and oxidative stress, *Drosophila* has been extensively utilized to evaluate antioxidants in vivo (Alam et al., 2013; Yi et al., 2021). Oxidative stress is characterized by overproduction of reactive oxygen species and reactive nitrogen species, that are collectively referred to as reactive species (RS). Imbalance between the RS production and impaired ability to detoxify RS or repair the damage results in oxidative stress. Oxidative stress can be induced by consumption of high calorie diet or ingestion of chemicals like Paraquat (Colpo et al., 2017; Trindade de Paula et al., 2016). Stress-induced inflammation and immune responses are also responsible for producing RS (Bayliak et al., 2019). These reactive species induce oxidation of biomolecules proteins, fatty acids and nucleic acids (Samet and Wages, 2018). Fruit flies possess three lines of conserved antioxidative defense mechanisms to prevent and repair damage induced by RS. (i) Antioxidative enzymes such as superoxide dismutase (SOD), catalase (CAT), glutathione peroxidase (GPx) and glutathione-S-transferase (GST) scavenge RS (Luo et al., 2020). (ii) Non-enzymatic antioxidants including reduced glutathione (GSH), ubiquinone and uric acid donate electrons to rapidly stop the radical oxidation reactions. (iii) The third and last line of defense includes enzymes that repair or remove the damaged biomolecules and include DNA repair enzymes, proteolytic enzymes, protein disulfide oxidoreductases and methionine sulfoxide reductase (Ighodaro and Akinloye, 2018; U and Ç, 2010). Food-derived antioxidants also contribute to the antioxidative response by (i) inactivating free radicals directly, (ii) by chelating and inactivating transition metals they protect organisms from oxidative damage and (iii) lastly, by upregulating expression or activity of antioxidative enzymes such as SOD, CAT, GST, GPx thioredoxin reductase (TRR) and glutathione reductase (GR) (Yi et al., 2021).

In this study we utilized *Drosophila melanogaster* as a model to test the effectiveness of biofortified wheat varieties in slowing down the ageing process. These anthocyanin rich blue, purple and black Indian wheat breeding lines with good yield potential were developed by cross-breeding and characterized for anthocyanin type and content by Garg et al. (Garg et al., 2016; Sharma et al., 2018). The black wheat lines had the highest anthocyanin content (185 ± 17.3 mg/kg), followed by blue wheat (113.3 ± 3.8 mg/kg), purple (40.7 ± 0.5 mg/kg) and amber/ white (2.2 ± 0.1 mg/kg) (Garg et al., 2016). Previous characterization of these native-colored wheat varieties in the mouse model indicates that incorporation of black colored wheat in the diet prevents high-fat diet induced obesity related metabolic complications (Sharma et al., 2020). Here, we have examined the long-term impact of these native-colored wheat varieties on *Drosophila* lifespan under different dietary contexts. Our data indicate that the biofortified blue wheat and not black wheat diet enhances lifespan in fruit flies exposed to *ad libitum* conditions. Gene expression analysis of RNA extracted from AL and DR-fed whole flies indicates that blue wheat formulated diet exerts its effects by modulating expression of *AMPK alpha*, *SREBP* and *Catalase* genes, suggesting that blue wheat-mediated antioxidant and DR mechanisms operate to extend lifespan in *Drosophila*. Blue wheat diet also enhanced survivability of fruit flies exposed to paraquat or high fat diet-induced oxidative stress and delayed degeneration of myofibrils in flies that were fed a high fat diet. In addition, blue wheat formulated diet provided resistance to oxidative stress in Sod2-deficient flies. Taken together, these data indicate that biofortified wheat formulated diets enriched in anthocyanin enhance healthspan and prevent cardiovascular age-related pathologies.

## 2 Materials and methods

### 2.1 Drosophila strains and husbandry

**Fig. 1:**
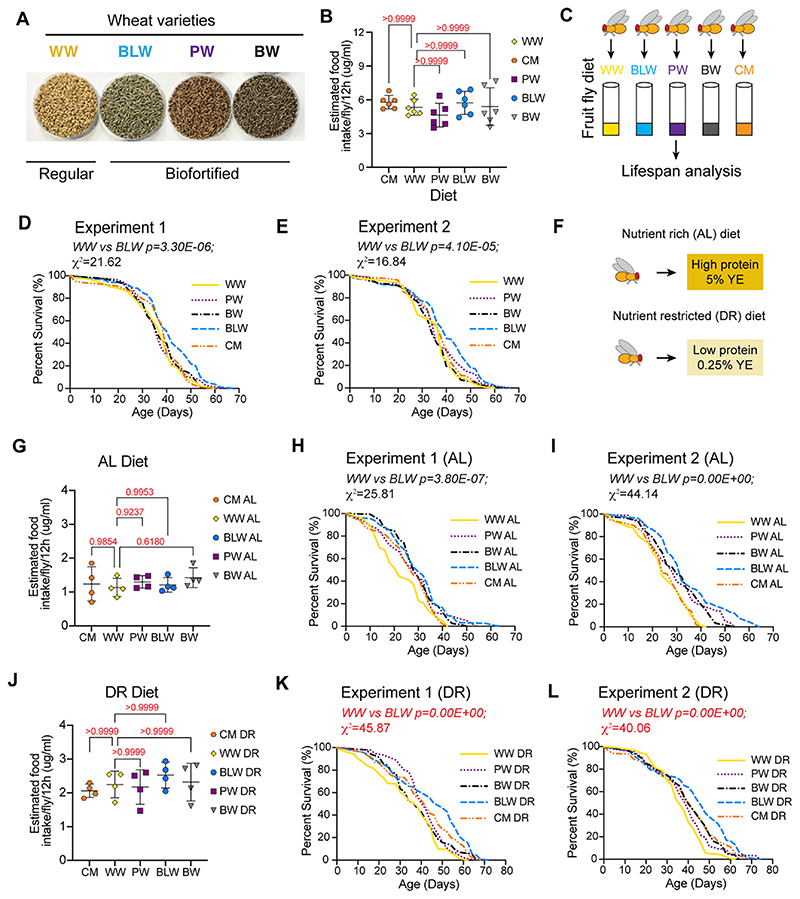
The effect of biofortified wheat on lifespan of adult *Canton S* female flies. (A) Different colored wheat varieties developed by conventional cross-breeding techniques. (B) Quantitation of food intake in flies that were fed different wheat-supplemented diets or the cornmeal diet. Estimated food intake on different diets (based on dye concentration) with 6 biological replicates of 10 flies each. Flies were fed on blue dye containing food for 12 h and transferred to food without dye for 3 h. Dye was collected from excreta solutions and quantified by measuring absorbance at 630 nm. Data are represented as mean ± SD, *n* = 6. *p*-Value calculated by ordinary one-way ANOVA is 0.3831. Adjusted *p*-values after applying Bonferroni's correction are represented in the figure and we used an α level of 0.05 to assess statistical significance. (C) Experimental design for lifespan analysis in diets prepared with cornmeal or wheat flour formulated diets. (D, E) Survival analysis of *Canton S* female flies that were fed the standard sugar-yeast (SY) medium containing 100% biofortified wheat flour substituted for cornmeal. (D) Survival curve for female *Canton S* flies that were fed a diet formulated with cornmeal (CM), white wheat flour (WW), purple wheat flour (PW), black wheat flour (BW) and blue wheat flour (BLW) in adulthood. The number of flies in each experiment were: white wheat (159), black wheat (140), blue wheat (160), purple wheat (160) and cornmeal (160). The BLW fed flies lived significantly longer than the WW fed flies (p-value after applying Bonferroni correction for WW versus BLW is 3.00E−05). (E) Survival curve for the second experimental repeat of female *Canton S* flies that were fed a diet supplemented with cornmeal (CM), white wheat (WW) or colored wheat flour (BW, PW or BLW). The number of flies in each experiment were: white wheat (140), black wheat (140), blue wheat (100), purple wheat (180), cornmeal (160). The BLW fed flies lived significantly longer than the WW fed flies (p-value after applying Bonferroni correction for WW versus BLW is 4.00E−04). (F) Scheme representing the experimental strategy for testing the effect of AL and DR diet on survivability of *Canton S* flies. AL diet contains 5% yeast extract and DR diet contains 0.25% protein yeast extract. (G–L)The effect of biofortified wheat on lifespan of adult *Canton S* female flies that were exposed to *ad libitum* (AL) or nutrient restricted (DR) diets. AL diet contains 5% yeast extract and DR diet contains 0.25% protein yeast extract. (G) Quantitation of food intake in flies that were fed different wheat-supplemented AL diets or the cornmeal AL diet. Estimated food intake on different diets (based on dye concentration) with 4 biological replicates of 10 flies each. Flies were fed on blue dye containing food for 12 h and transferred to food without dye for 3 h. Dye was collected from excreta solutions and quantified by measuring absorbance at 630 nm. Data are represented as mean ± SD, *n =* 4. *p*-Values calculated by ordinary one-way ANOVA is 0.4733. Adjusted *p*-values after applying Bonferroni's correction are represented in the figure and we used an α level of 0.05 to assess statistical significance. (H) Survival curve for female *Canton S* flies fed on biofortified wheat AL diet in adult stages. The number of flies in each experiment were: WW (124), BW (158), BLW (158), PW (136), CM (99). The BLW AL fed flies lived significantly longer than the WW AL fed flies (*p*-value after applying Bonferroni correction for WW AL versus BLW AL is 3.40E−06). (I) Survival curve for second experiment of female *Canton S* flies fed on biofortified wheat AL diet in adult stages. The number of flies in each experiment were - White wheat (115), Black wheat (126), Blue wheat (160), Purple wheat (160), Cornmeal (100). The BLW AL fed flies lived significantly longer than the WW AL fed flies (*p*-value after applying Bonferroni correction for WW AL versus BLW AL is 0.00E+00). (J) Quantitation of food intake in flies that were fed different wheat-supplemented DR diets or the cornmeal DR diet. Estimated food intake on different diets (based on dye concentration) with 4 biological replicates of 10 flies each. Flies were fed on blue dye containing food for 12 h and transferred to food without dye for 3 h. Dye was collected from excreta solutions and quantified by measuring absorbance at 630 nm. Data are represented as mean ± SD, *n =* 4. *p*-value calculated by ordinary one-way ANOVA is 0.6295. Adjusted *P*-values after applying Bonferroni's correction are represented in the figure and we used an α level of 0.05 to assess statistical significance. (K) Survival curve for female *Canton S* flies fed on biofortified wheat DR diet in adult stages. The number of flies in each experiment were - White wheat (139), Black wheat (140), Blue wheat (175), Purple wheat (136), Cornmeal (120). The BLW DR fed flies lived significantly longer than the WW DR fed flies (*p*-value after applying Bonferroni correction for WW DR versus BLW DR is 0.00E+00). (L) Survival curve for second experiment of female *Canton S* flies fed on biofortified wheat DR diet in adult stages. The number of flies in each experiment were- White wheat (138), Black wheat (126), Blue wheat (136), Purple wheat (160), Cornmeal (119). The BLW DR fed flies lived significantly longer than the WW DR fed flies (*p*-value after applying Bonferroni'scorrection for WW DR versus BLW DR is 0.00E+00). For statistical comparison of survival curves, *p*-values and χ ^2^ calculated with log rank test and *p*-values calculated after applying Bonferroni's correction are noted in the Tables 1A–1C. (For interpretation of the references to colour in this figure legend, the reader is referred to the web version of this article.)

**Fig. 2:**
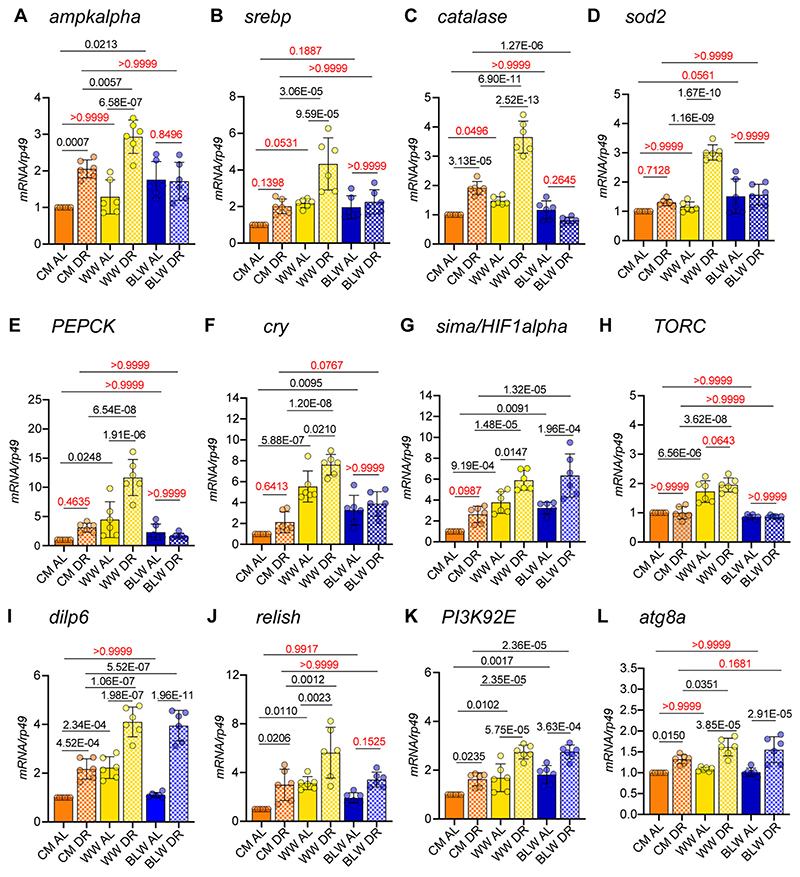
Effect of wheat formulated diet on the expression of age and DR-modulated genes. (A–L) Quantitative RT-PCR analysis of RNA extracted from *Canton S* female flies that were fed Cornmeal *Ad libitum* (CM AL) (orange bar), cornmeal DR diet (CM DR) (orange pattern bar), white wheat AL (WW AL) (yellow bar), white wheat DR (WW DR) (yellow pattern bar), blue wheat AL (BLW AL) (blue bar) and blue wheat DR (BLW DR) (blue pattern bar) for 10 days. (A) Bar graph representing expression of *ampkalpha* (*p*-value calculated by ordinary one-way ANOVA 9.10E−08). (B) Bar graph depicting expression of *srebp* (*p*-value calculated by ordinary oneway ANOVA 4.08E−07). (C) Quantitative RT-PCR of *catalase* (*p*-value calculated by ordinary one-way ANOVA <1.0E −15). (D) Bar graph representing expression analysis of *sod2* (*p*-value calculated by ordinary one-way ANOVA 3.05E−11). (E), Expression analysis of *PEPCK* (*p*-value calculated by ordinary one-way ANOVA 5.14E−10). (F), *cry* (*p*-value calculated by ordinary one-way ANOVA 2.58E−10). (G), *sima/HIF1alpha* (*p*-value calculated by ordinary one-way ANOVA 7.32E−09). (H), Quantitative RT-PCR analysis of *TORC* (*p*-value calculated by ordinary one-way ANOVA 2.57E−11). (I) Expression analysis of *dilp6* (*p*-value calculated by ordinary one-way ANOVA 2.30E−14). (J) Quantitative RT-PCR of *relish* (*p*-value calculated by ordinary one-way ANOVA 9.14E−07). (K) Quantitative RT-PCR of *PI3K92E* (*p*-value calculated by ordinary one-way ANOVA 9.94E−10). (L) Quantitative RT-PCR of *atg8a* (*p*-value calculated by ordinary one-way ANOVA 9.58E−08). Expression levels were normalized to *rp49.* Data are represented as mean ± SD, *n* = 3. Adjusted *p*-values after applying Bonferroni's adjusted *p*-values are represented in the figure and we used an α level of 0.05 to assess statistical significance. Genotypes of strains used in this figure: (A−L): *Canton S.* (For interpretation of the references to colour in this figure legend, the reader is referred to the web version of this article.)

**Fig. 3:**
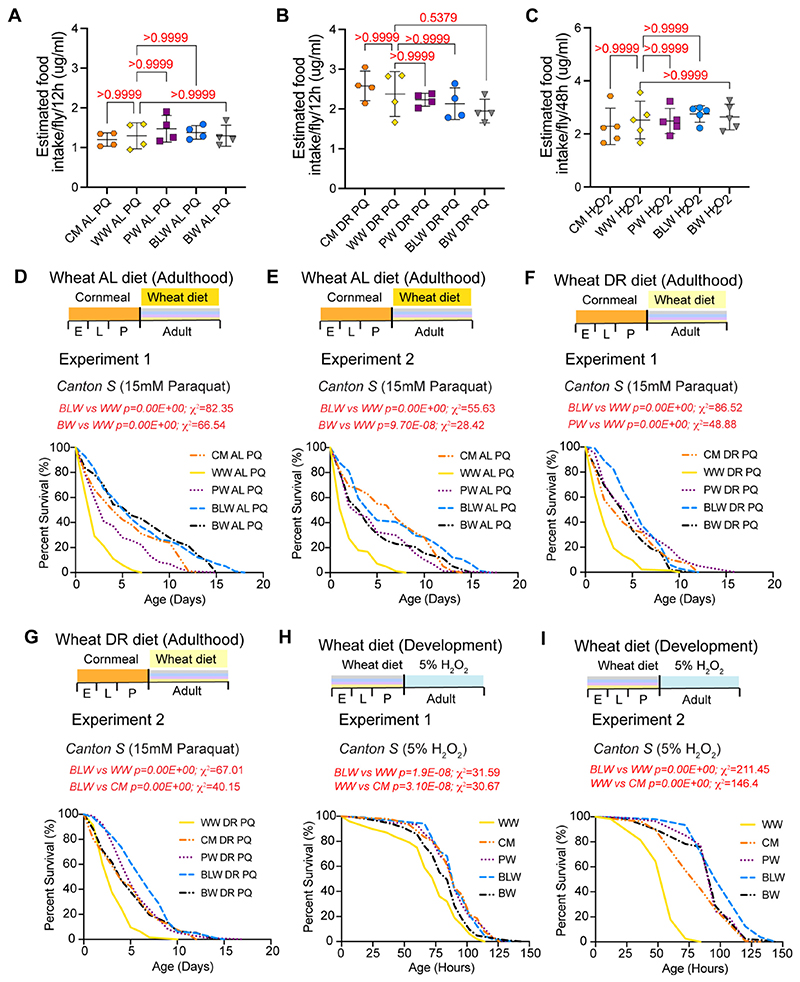
The effect of native wheat varieties and cornmeal formulated diet on paraquat-induced or hydrogen peroxide induced oxidative stress. (A−C) Quantitation of food intake in flies that were fed different *ad libitum* paraquat (AL PQ) diets (A), nutrient restricted paraquat (DR PQ) diets (B) or Hydrogen peroxide diets (C). Flies were fed on blue dye containing food for 12 h (A−B) or 48 h (C) and transferred to food without dye for 3 h. Dye was collected from excreta solutions and quantified by measuring absorbance at 630 nm. Data are represented as mean ± SD, *n =* 4 (A−B) and *n =* 5 (C). (A) *p*-Value calculated by ordinary one-way ANOVA is 0.6716. Adjusted *p*-values after applying Bonferroni's correction are represented in the figure panel and we used an α level of 0.05 to assess statistical significance. (B) *p*-Value calculated by ordinary one-way ANOVA is 0.2332. Adjusted *p*-values after applying Bonferroni's correction are represented in the figure and we used an α level of 0.05 to assess statistical significance. (C) *p*-Value calculated by ordinary one-way ANOVA is 0.7397. Adjusted *p*-values after applying Bonferroni's correction are represented in the figure and we used an α level of 0.05 to assess statistical significance. (D−E) Survival curve and dietary scheme for wild type *Canton S* flies that were fed different *ad libitum* (AL) wheat formulated diets with 15 mM paraquat (PQ) in adult stage and cornmeal diet during development (E, embryo; L, larval; P, pupal stage). Flies that were fed a BLW AL PQ diet were more resistant to paraquat than flies that were fed a WW AL PQ diet (For Experiments 1 and 2: *p*-value after applying Bonferroni's correction for WW versus BLW is 0.00E+00). (F−G) Dietary scheme and survival curve for female *Canton S* flies that were fed biofortified wheat DR diet with 15 mM paraquat in adult stages and cornmeal diet during development (E, embryo; L, larval; P, pupal stage). Flies that were fed a BLW DR PQ diet were more resistant to paraquat than flies that were fed a WW DR PQ diet (For Experiments 1 and 2: *p*-value after applying Bonferroni'scorrection for WW versus BLW is 0.00E+00). (H−I) Dietary scheme and survival curve for female *Canton S* flies that were reared on wheat diets during development (E, embryo; L, larval; P, pupal stage) and transferred to 5% Hydrogen peroxide in adult stages. Flies that were fed a BLW diet during development and hydrogen peroxide in adulthood were more resistant to paraquat than flies that were fed a WW diet during development and hydrogen peroxide during adulthood (Experiment 1: *p*-value after applying Bonferroni's correction for WW versus BLW is 7.70E−08; Experiment 2: *p*-value after applying Bonferroni's correction for WW versus BLW is 0.00E+00). For statistical comparison of survival curves, *p*-values and χ^2^ were calculated with log rank test and are noted in the Figure panels and Tables 2A–2C. Genotypes of strains used in this figure: (A−I) *Canton S.* (For interpretation of the references to colour in this figure legend, the reader is referred to the web version of this article.)

**Fig. 4:**
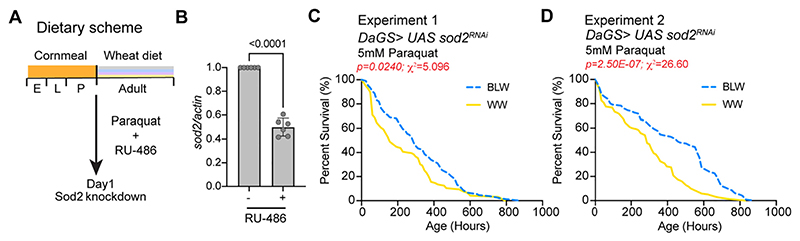
The effect of white and blue wheat formulated diet on paraquat-induced oxidative stress in *Sod 2* deficient flies. (A) Dietary scheme for analysis of *daGS > UAS SOD^RNAi^* flies that were fed 5 mM paraquat in white or blue wheat based diets during adulthood and cornmeal based diet during development (E, embryonic stage; L, larval stage; A, adult stage). (B) A transgene expressing dsRNA for RNAi of *Sod2* was expressed ubiquitously using the steroid (RU-486) inducible gene switch Daughterless Gal4 driver. Quantitative RT-PCR of *sod2* with RNA extracted from *daGS > UAS Sod2^RNAi^* female flies in presence or in absence of RU-486 for 5 days. Expression levels were normalized to *actin.* Data are represented as mean ± SD, *n =* 3. Two technical replicates for each biological replicate (*n* = 3) were used for the analysis. *p*-Value was calculated using unpaired *t*-test with Welch's correction and is noted in the bar graph. (C) Survival curve for female *daGS* > *UAS Sod2^RNAi^* flies that were fed biofortified wheat with 5 mM paraquat in adult stages. The number of flies in each experiment were: White wheat (96), Blue wheat (106). (D) Survival curve for second trial of female *daGS* > *UAS Sod2^RNAi^* flies that were fed biofortified wheat with 5 mM paraquat in adult stages. The number of flies in each experiment were - White wheat (120), Blue wheat (106). For statistical comparison of survival curves, *p*-values and χ ^2^ were calculated with log rank test and are noted in the Figure panels and Table 2D. Genotypes of strains used in this figure: (A−D) *DaGS* > *UAS Sod2^RNAi^:* +/+;P{y[+t7.7] v [+t1.8] = TRiP.GL01015}attP40/. P{w[+mW·hs] = Switch1}DaGS. (For interpretation of the references to colour in this figure legend, the reader is referred to the web version of this article.)

**Fig. 5:**
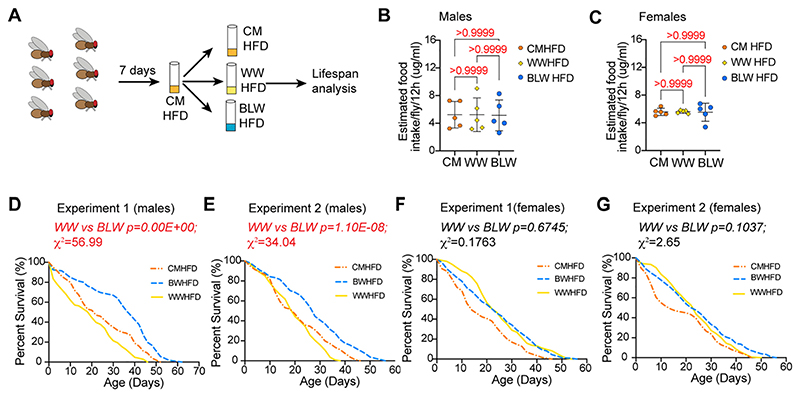
The effect of biofortified wheat on lifespan of adult *Canton S* female flies that were fed a high fat diet (HFD). (A) Schematic representation of the experimental strategy. *Canton S* flies were maintained in cornmeal food during development (E, Embryo; L, larval stage and P, pupal stage) and 2−3 day old mated adult flies were sorted into male and females and transferred to a cornmeal formulated high fat diet (CMHFD) for 7 days prior to transfer into white wheat flour formulated high fat diet (WWHFD) or blue wheat flour formulated high fat diet. Survivability of males or females was measured in CMHFD, WWHFD or BLWHFD. (B) Quantitation of food intake in male flies that were fed WWHFD, BLWHFD or the CMHFD. Estimated food intake on different diets (based on dye concentration) with 5 biological replicates of 10 flies each. Flies were fed on blue dye containing food for 12 h and transferred to food without dye for 3 h. Dye was collected from excreta solutions and quantified by measuring absorbance at 630 nm. Data are represented as mean ± SD, *n* = 5. *p*-Value calculated by ordinary one-way ANOVA is 09971. Adjusted *p*-values after applying Bonferroni's correction are represented in the figure and we used an α level of 0.05 to assess statistical significance. (C) Quantitation of food intake in female flies that were fed WWHFD, BLWHFD or the CMHFD. Data are represented as mean ± SD, *n* = 5. *p*-Value calculated by ordinary one-way ANOVA is 09904. Adjusted *p*-values after applying Bonferroni's correction are represented in the figure and we used an α level of 0.05 to assess statistical significance. (D) Survival curve for experiment 1 with male *Canton S* flies fed on CMHFD, WWHFD or BLWHFD. The number of flies in each group were: WWHFD (103), BLWHFD (106), and CMHFD (100). The median lifespan in: CMHFD was 22 days; WWHFD was 20 days and BLWHFD was 38 days. *p*-Value calculated after applying Bonferroni correction: WWHFD vs BLWHFD = 0.00E+00. (E) Survival curve for experiment 2 with male *Canton S* flies fed on WWHFD, BLWHFD or CMHFD. The number of flies in each experiment were: WWHFD (113), BLWHFD (108), and CMHFD (81). The median lifespan in CMHFD and WWHFD was 20 days and in BLWHFD was 28 days. *p*-Value calculated after applying Bonferroni correction: WWHFD vs BLWHFD = 1.10E−08. (F) Survival curve for experiment 1 with female *Canton S* flies fed on CMHFD, WWHFD or BLWHFD. The number of flies in each experiment were: WWHFD (145), BLWHFD (137), CMHFD (118). The median lifespan in CMHFD was 14 days, WWHFD was 24 days and BLWHFD was 24 days. *p*-Value calculated after applying Bonferroni correction: WWHFD vs BLWHFD = 1. (G) Survival curve for second experiment with female *Canton S* flies fed on CMHFD, WWHFD or BLWHFD. The number of flies in each experiment were: WWHFD (165), BLWHFD (126), and CMHFD (109). The median lifespan in CMHFD was 14 days; WWHFD was 24 days and BLWHFD was 22 days. *p*-Value calculated after applying Bonferroni correction: WWHFD vs BLWHFD = 0.5187. For statistical comparison of survival curves, *p*-values and χ ^2^ were calculated with log rank test and after applying Bonferroni correction and are listed in Table 3. Genotypes of strains used in this figure: (A−G) *Canton S*. (For interpretation of the references to colour in this figure legend, the reader is referred to the web version of this article.)

**Fig. 6:**
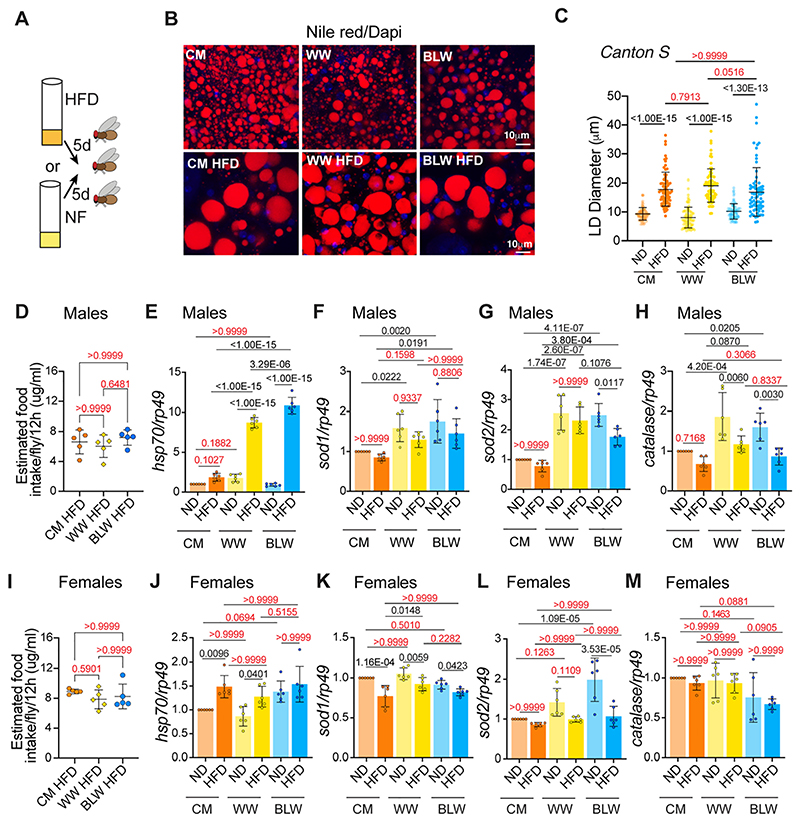
Effect of wheat formulated high fat diet on expression of genes involved in oxidative stress. (A) Schematic of the dietary regimen used for gene expression analysis. 2−3-day old mated wild type *Canton S* flies were transferred to normal diet (ND) or high fat diet (HFD) formulated with cornmeal (CM), white wheat (WW) or blue wheat (BLW). (B) Fat bodies/abdomens of 5-day old flies were dissected and stained for the content and diameter of lipid droplet (LD) (red are lipid droplets stained with Nile red and blue is Dapi). Scale bar, 10 μm. (C) Quantitation of lipid droplet (LD) diameter in (B). Quantitation of 15 largest LDs in 5 samples per condition. Error bars represent mean ± SD and *p*-value calculated by ordinary one-way ANOVA is <1.00E−15. Adjusted *p*-values after applying Bonferroni's correction are represented in the figure and we used an α level of 0.05 to assess statistical significance. A significant increase in lipid droplet size was seen in the fat tissue of flies that were fed CMHFD, WWHFD or BLWHFD relative to CM, WW or BLW diet. (D) Quantitation of food intake in male flies that were fed WWHFD, BLWHFD or the CMHFD. Data are represented as mean ± SD, *n* = 5. *p*-Value calculated by ordinary one-way ANOVA is 0.4505. Adjusted *P*-values after applying Bonferroni's correction are represented in the figure and we used an α level of 0.05 to assess statistical significance. (E−H) RT-PCR quantitation of *hsp70*, *sod1*, *sod2 and catalase* mRNA in whole male *Canton S* flies that were fed a normal food (NF) or high fat diet (HFD) for 5 days. Expression levels were normalized to *rp49*. Values are mean ± SD, *n* = 3. For each biological replicate (*n* = 3), two technical replicates were analyzed. (E) HFD significantly induces expression of *hsp70* in male files that were fed wheat diets than in CM diet. *P*-value calculated by ordinary one-way ANOVA is <0.0001. Adjusted *p*-values after applying Bonferroni's correction are represented in the figure and we used an α level of 0.05 to assess statistical significance. (F) *Sod1* was expressed at higher levels in *Canton S* flies that were fed wheat-supplemented diets as compared to CM diet and did not vary significantly between wheat HFD or CM HFD. *p*-Value calculated by ordinary one-way ANOVA is 0.0001. Adjusted *P*-values after applying Bonferroni's correction are represented in the figure and we used an α level of 0.05 to assess statistical significance. (G) *Sod 2* was expressed at higher levels in *Canton S* flies that were fed wheat-supplemented normal diets or HFD as compared to CM and CMHFD. *p*-Value calculated by ordinary one-way ANOVA is 2.00E−10. Adjusted *p*-values after applying Bonferroni's correction are represented in the figure and we used an α level of 0.05 to assess statistical significance. (H) *Canton S* flies that are fed WW or BLW diets express significantly higher levels of Catalase than flies that are fed CM diet. *p*-Value calculated by ordinary one-way ANOVA is 1.66E−06. Adjusted *p*-values after applying Bonferroni's correction are represented in the figure and we used an α level of 0.05 to assess statistical significance. (I) Quantitation of food intake in female flies that were fed WWHFD, BLWHFD or the CMHFD. Data are represented as mean ± SD, *n* = 5. *p*-Value calculated by ordinary one-way ANOVA is 0.4116. Adjusted P-values after applying Bonferroni's correction are represented in the figure and we used an α level of 0.05 to assess statistical significance. (J−M) RT-PCR quantitation of *hsp70, sod1, sod2 and catalase* mRNA in whole female *Canton S* flies that were fed a normal food diet (ND) or high fat diet (HFD) for 5 days. (J) Canton S female flies that are fed a blue wheat diet express significantly higher levels of *hsp70* than flies that are fed a CM or WW diet. However, female flies that are fed CMHFD, WWHFD or BLWHFD express similar levels of *hsp70*. *p*-Value calculated by ordinary one-way ANOVA is 7.28E−05. Adjusted *p*-values after applying Bonferroni's correction are represented in the figure and we used an α level of 0.05 to assess statistical significance. (K) Expression of *Sod1* does not differ significantly in *Canton S* female flies that were fed a CM, WW or BLW diet. *p*-Value calculated by ordinary one-way ANOVA is 3.07E−06. Adjusted *p*-values after applying Bonferroni's correction are represented in the figure and we used an α level of 0.05 to assess statistical significance. (L) *Canton S* females that were fed a BLW diet or WW diet expressed significantly higher levels of *sod2* as compared to flies that were fed a CM diet. Supplementing the diet with fat reduced expression of *sod2* in all the diets. *p*-Value calculated by ordinary one-way ANOVA is 8.85E−07. Adjusted*p*-values after applying Bonferroni's correction are represented in the figure and we used an α level of 0.05 to assess statistical significance. (M) Catalase was expressed at similar levels in *Canton S* female flies that were fed CM, WW or BLW diets. *p*-Value calculated by ordinary one-way ANOVA is 0.0107. Adjusted *p*-values after applying Bonferroni's correction are represented in the figure and we used an α level of 0.05 to assess statistical significance. Expression levels were normalized to *rp49*. Values are mean ± SD, *n* = 3. For each biological replicate (*n* = 3), two technical replicates were analyzed. Genotypes of strains used in this figure: (A−M) *Canton S.* (For interpretation of the references to colour in this figure legend, the reader is referred to the web version of this article.)

**Fig. 7:**
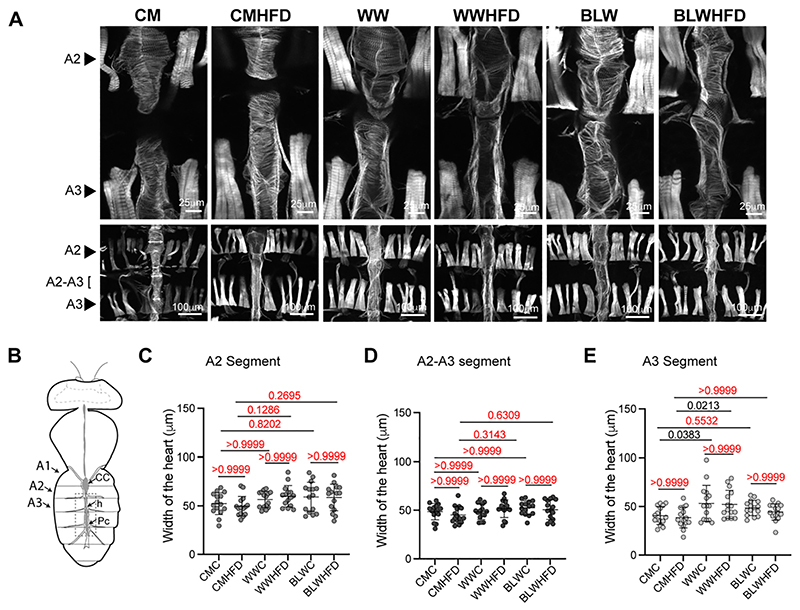
Effect of wheat formulated high fat diet on *Drosophila* heart structure. (A) Fluorescent micrographs of the dissected abdomen of wild type *Canton S* flies that were fed a corn meal (CM), white wheat (WW) or blue wheat (BLW) flour formulated normal diet or high fat diet (HFD). Flies were fed CM, WW or BLW-supplemented diets throughout development and 2−3 days post-eclosion, the flies were shifted to CM, WW or BLW-formulated HFD for 27 days. Adult hearts were stained with Alexa fluor 594 phalloidin. The images were taken at 20× (upper panel) and 10× (lower panel) magnification. The adult heart has circum-ferentially oriented myofibrils that are compactly arranged in flies that are fed a normal diet. Disorganization of the myofibrillar structure was detected in the tissue of flies that were fed a high fat diet, with WWHFD samples displaying the most severe defects compared to the tissue from CMHFD and BLWHFD fed flies. (B) Schematic of the dissected abdomen with the main conical chamber (CC), heart (h), located dorsally along the abdominal segments 1 (A1). Pericardial cells (Pc) are closely associated along the length of the abdominal portion of the circulatory system and are noncontractile cells that are implicated in detoxification of the hemolymph. (C−E) Quantitation of the diameter of the heart tube in (C) A2 segment (*p*-value calculated by ordinary one-way ANOVA is 0.1057). Adjusted *p*-values after applying Bonferroni's correction are represented in the figure and we used an α level of 0.05 to assess statistical significance. (D) Region in between A2 and A3 segment (*p*-value calculated by ordinary one-way ANOVA is 0.2217). Adjusted *p*-values after applying Bonferroni's correction are represented in the figure and we used an α level of 0.05 to assess statistical significance and (E) A3 segment (*p*-value calculated by ordinary one-way ANOVA is 0.0044). Adjusted *p*-values after applying Bonferroni's correction are represented in the figure and we used an α level of 0.05 to assess statistical significance. Quantitation of the heart tube (3 regions per segment) in 5 samples per condition. Error bars represent mean ± SD. Genotypes of strains used in this figure: (A−E) *Canton S.* (For interpretation of the references to colour in this figure legend, the reader is referred to the web version of this article.)

*Drosophila melanogaster* stocks of the wildtype strain *Canton S* and *w^1118^* have been in the laboratory since 2017 and have been maintained in sugar-yeast cornmeal-based diet (please see Section 2.2 for recipe). The flies have been maintained in 500 mL glass bottles or vials by transferring the new generation of flies to fresh food bottles or vials. *Canton S* flies were used for experiments depicted in Fig. 1, Fig. 2, Supplementary Fig. 1, Fig. 3, Fig. 5, Fig. 6, and Fig. 7. The *UAS-Sod2^RNAi^* line (BL# 36871) and *Da-GS* Gal4 (gift from David Walker's laboratory) driver were used for the experiments in Fig. 4 and Supplementary Fig. 2. These lines were backcrossed three times into a homogenous control background *w^1118^* by setting up crosses with a single male from each line with three female *w^1118^* virgin flies. Single F1 progeny males that harbored the transgene were scored and selected based on the expression of the white gene and crossed with three female *w^1118^* virgin flies. The F2 progeny males were scored and selected as described for F1 progeny and crossed again with three female virgin *w^1118^* flies. The process was repeated with F3 males and the F4 male progeny were crossed with a compound chromosome 2:3 lab balancer stock. The resulting balanced stocks were used for setting up crosses for lifespan analysis. All flies used in the study were maintained in standard corn-meal/agar medium at 25 °C with a 12 h light: 12 h dark cycle in 60% humidity. For steroid mediated *UAS-Sod2^RNAi^* control using the Gene-Switch driver, flies were fed a diet containing 200 μM RU-486 (Mife-pristone, Cayman Chemicals, Ann Arbor MI). Comparisons of lifespan were made in the same strain in different diets in parallel. The lack ofany effect of RU-486 on *Da-GS* has been reported previously(Bolukbasi et al., 2017). The lack of any effect of ligand/RU-486 on *UAS Sod^RNAi^* strain was confirmed and represented in Supplementary Fig. 2. Unless otherwise noted, all assays utilized adult mated (48 h) female flies of indicated ages. All the experiments were performed at 25 °C apart from the survival analysis of *Canton S* in diets formulated with coconut oil that were performed at 20 °C.

### 2.2 Preparation of Drosophila diets

*Cornmeal/agar food* (NF): The cornmeal/agar food was prepared by adding cornmeal (86 g), sucrose (25 g), Dextrose (51 g), Yeast extract (15 g), Agar (4.6 g), 1% Acid mix (10 mL) and Tegosept (5 mL; 1 g of Methyl 4-hydroxybenzoate [SRL] in 5 mL 100% Ethanol) per 1000 mL of food. The acid mix stock solution was prepared by combining propionic acid (164 mL autoclaved milliQ water with 836 mL propionic acid [SRL]) and orthophosphoric acid (917 mL autoclaved milliQ water to 83 mL of orthophosphoric acid [SRL]). The wheat/agar food was prepared by substituting wheat for cornmeal in this protocol.

*Ad libitum* (*AL*) *and dietary restricted* (*DR*) *food*: *Ad libitum* and dietary restricted food was prepared to observe the effect of protein restriction in the diet and 80% wheat flour, and 20% cornmeal was used as a carbohydrate source. AL food was prepared by adding Yeast Extract (50 g), Sucrose (50 g), wheat (68.8 g), Cornmeal (17.2 g), Agar (4.3 g), 1% Acid mix (10 mL) and Tegosept (5 mL; 1 g in 5 mL 100% Ethanol) per 1000 mL food. To restrict protein concentration, yeast extract was restricted in DR food (2.5 g/1000 mL food), rest other components remained same.

*Paraquat Food*: Methyl viologen dichloride, Paraquat (Sigma Aldrich) was used to study the ability of flies fed on antioxidant rich wheat food to resist the oxidative stress induced. To make 15 mM Paraquat solution 192 mg of Paraquat was weighed and dissolved in 500 μL water. Fifty milliliters Wheat AL and DR food was melted and cooled down to 40 °C. Paraquat solution was added to the melted food. It was mixed evenly by stirring and layered 2 mL each on 1.5% Agar vials.

*High Fat diet (HFD)*: HFD was prepared by adding 25% (for quantitative real time PCR analysis) or 30% (for lifespan assays and phalloidin staining) coconut oil to regular fly food. Twenty-five grams (for 25%) or 30 g (for 30%) coconut oil was melted and added to 75 g (for 25%) or 70 g (30%) of regular fly food, respectively. A homogenous mixture was made by continuous stirring of the components. Five milliliters per vial was aliquoted. For lifespan experiments, flies were fed HFD and Normal Food (NF) for 7 days and after 7 days flies were transferred to different wheat based high fat diets. For phalloidin staining, flies were fed CM, WW or BLW-supplemented diets throughout development and 2 days post-eclosion, the flies were shifted to CM, WW or BLW-formulated HFD for 27 days. Vials were kept horizontally to prevent sticking of flies to the food. For quantitative RT-PCR in Figs. 6, 2 days aged mated flies were transferred to HFD or NF diet for 5 days prior to preparation of the RNA.

### 2.3 Measurement of food intake

The Excreta measurement (EX-Q) method was utilized to measure food intake in flies that were fed the different wheat diets(Wu et al., 2020). Ex-Q tubes were made from 14 mL round bottom tubes (ThermoFisher Scientific 150268). The cap of the tube has an inbuilt ~6 mm cavity and that was used as a food container. Six airholes were made in the cap around the cavity using a pushpin. For wheat and cornmeal food used in EX-Q experiments 1% agarose (Seakem LE agarose, Lonza 50004) was used for preparation instead of agar. The Erioglaucine (SRL 98188, for dye food) was added to the medium after cooling to 60 °C.

Three-day old adult mated female or male flies were acclimatized to the experimental food for 48 h for EX-Q assays in Figure panels 1B, 1G and 1 J. For EX-Q measurement in paraquat and HFD (Figure panels 3A, 3B, 3C, 5B, 5C, 6D and 6I) the three-day old flies were acclimatized for 24 h as 48 h exposure to these agents leads to death of some flies. For EX-Q measurement in H_2_O_2_ *Canton S* flies were reared on the different diets to be tested and three-day old flies were acclimatized on H_2_O_2_ medium for 24 h. Different wheat or cornmeal medium containing 2.5% (*w*/*v*) Erioglaucine was used as the assay medium. Absorbance of solubilized excreta (1000 μL) was measured at 630 nm (Molecular Devices, Spec-traMax i3x) in a 96-well plate. The food intake per fly was calculated from a standard curve prepared from stock solutions of pure dye (0.01–0.065 mg/mL). The absorbance of 200 μL sample was measured at 630 nm. A minimum of 4 replicates of 10 flies each were used for assays and individual data points were plotted in each panel and the number of replicates were noted in the figure legends. The blank calculations were performed by preparing homogenates from age-matched flies that were fed a food that was lacking Erioglaucine.

### 2.4 Lifespan analysis

The Kaplan-Meier method was utilized to generate survival curves for all lifespan experiments using the *Online Application for the Survival Analysis of lifespan assays* (OASIS) and GraphPad prism and the *p*-values were calculated using the log-rank (Mantel-cox) test and for experiments where multiple comparisons were made *p*-values were also calculated after applying Bonferroni's correction and are noted in the Tables 1A–1C, Tables 2A–2C and Table 3 (Han et al., 2016; Yang et al., 2011). All experiments were performed with flies that were allowed 48 h to mate after emerging as adults. On the third day after eclosion, flies were anesthetized with carbon dioxide, sorted, counted, and distributed between different nutrient regimes for experiments. Around 20 flies were transferred to each vial. For longevity assay live flies were transferred to fresh medium every third day and counted every alternate day until no flies remained.

For measurement of resistance to Hydrogen peroxide, *Canton S* flies were reared in different wheat-based diets throughout development and newly eclosed 2 day aged mated female flies were shifted to 5% H_2_O_2_ to check the efficacy of different wheats in providing resistance to oxidative stress. To make H_2_O_2_ vials 10 g sucrose and 0.8 g agarose were added to 10 mL 10× PBS. 73.4 mL water was added to it and heated until a clear liquid was formed. Solution was cooled down to 40 °C. 16.6 mL of 30% H_2_O_2_ (Sigma Aldrich) was added to the sucrose agar solution and 10 mL was aliquoted in each vial.

### 2.5 Immunostaining and microscopy

#### 2.5.1 Nile red staining

Abdomens of adult flies were dissected in 1× phosphate buffered saline (PBS) and fixed for 30 min in 4% paraformaldehyde in PBS at room temperature. The fixed samples were rinsed three times with 1× PBS + 0.3% Triton (PBST). Tissues were stained for 1 h with Nile Red solution (1 mg/mL in Acetone,1:250 PBST) and DAPI (1:100 PBST). Three washes of 5 min each were given with PBS-T and samples were mounted. The lipid droplet diameter was measured in ImageJ software and graphs were plotted with GraphPad Prism v8.

#### 2.5.2 Phalloidin staining

For the staining of the heart, the abdomens of adult flies were dissected in 1× PBS and fixed in 4% PFA for 30 min. The fixed tissues were washed three times for 5 min each were given in 1× PBS + 0.3% Triton. Tissues were stained overnight with Phalloidin (1:50 PBST) and DAPI (1:100 PBST). The samples were rinsed three times in PBST for 5 min each and mounted in 90% Glycerol-n-Propyl gallate. All experiments were imaged on the Leica SP8 confocal microscope. For phalloidin staining images were acquired at 10× and 20× magnification.

### 2.6 RNA isolation and quantitative real time PCR

Total RNA was extracted from 3 to 5 adult flies using RNAiso Plus (Takara Bio, Inc). Animals were homogenized in 0.2 mL of RNAiso Plus with a white micropestle (Tarsons) prior to extraction. The cDNA was generated by using a High-capacity cDNA reverse transcription Kit (Thermo Fisher Scientific, MA, USA). In each reaction 0.5–1 μg was mixed with random hexamers, MgCl_2_, 10× RT Buffer, dNTPs, RNAse Inhibitor and MultiScribe Reverse transcriptase in a 10 μL total volume. The cDNA synthesis was performed as per manufacturer's protocol in a Bio-Rad C1000 Touch Thermal Cycler. The synthesized cDNA was diluted (1:10) and used as template for quantitative real-time PCR (qRT-PCR) using SYBR premix EX-tAQ-plus (Takara Bio, Inc) and analyzed on QuantStudio 6 Real-Time PCR machine (Applied Biosystems, Foster City, CA, USA). The expression of the target genes was normalized to *actin-5C* or *rp49.* All RT-PCR data were generated with three independent biological replicates (different RNA extractions) and two technical replicates for each biological replicate.

Primers used for RT-PCR are: act-5c For, cacaccaaatcttacaaaatgtgt; act-5c Rev., aatccggccttgcacatg; rp-49 For, cccaagggtatcgacaacaga; rp49 Rev., cgatgttgggcatcagatactg; AMPK alpha For, agaggtctgcac-caagttcg; AMPK alpha Rev., SREBP For, caccgacaagcacaggtagt; SREBP Rev., gatcgtgattctggcgttgc; Sod 2 For, agaacctctcgcccaacaag; Sod 2 Rev., actgcagatagtaggcgtgc; Catalase For, caaccccttcgatgtcacca; Catalase Rev., catcctggttgtccgtcaca; Pepck For, gctggccaagaaggaggaat; Pepck Rev., cgtagcccgatccgtaagac; TORC For, atttcgtggccctcgacttt; TORC Rev., agtccaacatctccggcttg; Sima/Hif1alpha For, agcggcaaaccaaaggagaa; Sima/Hif1alpha Rev., ccgttgagccaatcctcagt; Cry For, ccgttctctatgtcgg-gagc; Cry RT PCR Rev., gatcgtggacgctccagtag; Rel For, tccttttcaccagcc-cactc; Rel Rev., ggcacgtgtttgatatcgcc; Dilp 6 For, cgatgtatttcccaacagtttcg; Dilp 6 Rev., aaatcggttacgttctgcaagtc; Pi3K92E For, acctaatctgcctgttgccc; Pi3K92E Rev., actgagtcgcttcgtttcgt; Atg8a For, cgtcgcaaatatccagaccg; Atg8a Rev., gaagaagagggcatcctcgg; Sod1 For, caagggcacggttttcttcg; Sod1 Rev., tacggattgaagtgcggtcc; HSP70 For, tggacaagtgcaacgacact; HSP70 Rev., tcgatcgaaacattcttatcagtct; PRAS40 For, agcctcagcacaatcccttc; PRAS40 Rev., gccatggacacttctggtca; Mitf For, cgtgagggccatccaaatca; Mitf Rev., cttcgtctacggtgccaagt; Nrf2 For, tgga-catgttagcaacggct; Nrf2 Rev., gatggtaatcctgggcggag.

### 2.7 Statistical analyses

Statistical analysis and data presentation was performed with GraphPad Prism 8 software, OASIS and Microsoft Excel. Survival curves were compared using log-rank tests and in figures where multiple comparisons were made *p*-values were calculated after applying Bon-ferroni's correction and noted in the figure legends and Tables. All survival and lifespan graphs show two repeats with cohorts of 20 female flies per genotype. An ordinary one-way ANOVA was used to analyze data from RT-PCR, EX-Q and diameter measurement of heart tube where multiple comparisons were made. All RT-PCR analysis was performed with three independent biological replicates and two technical replicates for each biological replicate and individual data points were plotted in all graphs. Adjusted *p*-values for all comparisons were computed by applying Bonferroni's correction and noted in the figure panels. The significant *p*-Values were denoted in black font and the non-significant values were noted in red font. Statistical significance was set at *p* < 0.05.

## 3 Results

### 3.1 Effect of biofortified wheat flour formulated diet on the survivability of wild type flies

Biofortified cereals such as wheat enriched in anthocyanin content prevent high fat diet-induced obesity in the mouse model (Sharma et al., 2020). However, the effectiveness of these wheat varieties in modulating lifespan has not been reported in mouse or any other animal model. To determine whether the biofortified wheat flour varieties influenced survivability we measured lifespan of wild type *Drosophila melanogaster* flies that were fed a diet formulated with the colored biofortified wheat flour. A diet formulated with the traditional white wheat flour was used as control for examining the effects of blue, purple and black crossbred wheat varieties. In parallel, we also examined the effects of the standardized cornmeal diet that has been traditionally used for rearing *Drosophila* in several laboratories. *Canton S* flies were reared on cornmeal diet and were transferred to wheat flour formulated diets in adulthood (Fig. 1A). To examine whether the wheat-based diets were ingested to comparable levels, the quantity of food ingested was measured by an Excreta quantitation assay (EX-Q) (Wu et al., 2020). Ten flies (per replicate) were transferred to an EX-Q chamber and fed a diet containing 2.5% Erioglaucine dye in a small lid. After 12 h of feeding the dye medium was replaced with food lacking the dye for 3 h to allow the flies to excrete almost all residual dye containing food in the body. The excreta were recovered from the tube walls and cap by removing the flies from the tube and rinsing with MilliQ water and quantitated by measuring absorbance at 630 nm. No significant difference was seen in the food ingested by *Canton S* flies that were fed a WW, CM, BLW, PW or BW-supplemented diet (*p*-value calculated by ordinary one-way ANOVA test is 0.3831) (Fig. 1B). Next, we measured survival of flies that were fed different diets. The adult flies were cultured in either cornmeal (CM), white wheat flour (WW), purple wheat flour (PW), black wheat (BW) or blue wheat (BLW) flour diet and experiments were carried out in vials containing 20 flies each (Fig. 1C). Wild type (*Canton S*) flies that were fed a blue wheat flour-supplemented diet (blue line) (BLW) lived significantly longer than wild type flies that were fed a white wheat (WW) (yellow line) flour-supplemented diet (Experiment 1: *p* = 3.30E−06; χ^2^ = 21.62; Experiment 2: *p =* 4.10E−05; χ^2^ = 16.84) (Fig. 1D–E). The median lifespan of BLW fed flies was 5–10% longer than WW fed flies (Table 1A). *Canton S* flies that were fed either black (BW) or purple (PW) wheat or the standard cornmeal (CM) formulated diet did not display a statistically significant increase in lifespan extension when compared with flies that were fed the WW diet (Fig. 1D–E) (Table 1A). Taken together, these data confirmed that diet formulated with biofortified wheat varieties influenced lifespan differentially and the increase in longevity did not correlate with the amount of anthocyanin content as flies that were fed a diet with black wheat (BW) that has been previously reported to have the highest anthocyanin did not live longer than the flies that were fed a blue wheat (BLW) formulated diet. However, biofortified blue wheat flour diet significantly enhanced lifespan of wild type flies when compared with flies that were fed white wheat flour or cornmeal formulated diet.

### 3.2 Effect of wheat flour formulated diet on the survivability of wild type flies that were fed ad libitum (AL) or nutrient restricted (DR) diet

Dietary restriction (DR) or reduction (chronic or intermittent) in food intake without malnutrition, represents one of the most conserved environmental interventions to extend lifespan and attenuate.

age-related diseases (Fontana and Partridge, 2015; McCay et al., 1989). Restriction of calorie intake or caloric restriction (CR) is one form of DR that has also been shown to extend lifespan in a wide range of animals and improve metabolic health parameters in humans (Lin et al., 2004). One mechanism that has been proposed for an increase in life-span upon caloric restriction (CR) is the decrease in oxidative stress and increase in antioxidant defense (Meydani et al., 2011; Sohal and Weindruch, 1996; Wanagat et al., 1999). High fiber cereal diets have been shown to facilitate CR in humans (Gilhooly et al., 2008), hence, we examined the effects of biofortified wheat formulated AL (Fig. 1G–I) and DR (Fig. 1J–L) diets on *Drosophila* lifespan. DR imposed by restricting protein content in the diet has been shown to significantly enhance lifespan of rodents, flies, and yeast (Mair et al., 2005; Min and Tatar, 2006; Minor et al., 2010; Mirisola et al., 2014; Pandey et al., 2021). Hence, we used the dietary regimen in which the AL and DR diets differed in the protein content. Since female flies are more responsive to DR in *Drosophila*, we measured the effect of wheat formulated AL and DR diets in modulating lifespan in female fruit flies (Magwere et al., 2004). To examine whether the wheat-based AL and DR diets were ingested to comparable levels, the quantity of food ingested was measured by an Excreta quantitation assay (EX-Q) (Wu et al., 2020). No significant difference was seen in the food ingested by *Canton S* flies that were fed a WW AL, CM AL, BLW AL, PW AL or BW AL diet (*p*-value calculated by ordinary one-way ANOVA = 0.4733) (Fig. 1G) or WW DR, CM DR, BLW DR, PW DR or BW DR (*p*-value calculated by ordinary one-way ANOVA = 0.6295) (Fig. 1J). To examine the impact of colored wheat formulated AL diets on lifespan of wild type flies, survival analysis was performed with *Canton S* flies that were fed AL and DR diets formulated with CM, WW, PW, BLW and BW (Fig. 1E–I). Wild type flies that were fed the BW AL, BLW AL or PW AL diet lived significantly longer than wild type flies that were fed a WW formulated AL diet (yellow solid line). BW AL fed flies had a 20–21% increase in median lifespan, BLW AL fed flies had a 25–26% longer median lifespan and PW AL fed flies had a 14–21% longer median lifespan as compared to WW AL fed flies, respectively (Fig. 1F–G) (Table 1B). Wild type flies that were fed a BW DR, BLW DR or PW DR diet lived significantly longer than wild type flies that were fed a WW DR diet (yellow dashed line) (Fig. 1H–I). Compared to *Canton S* flies that were fed a WW DR diet, BW DR fed flies had a minimal increase in median life span (0–10%) followed by PW DR (9.5–10%) and greatest increase was seen when flies were fed a BLW DR (21–22%) diet (Table 1C). Taken together, these data indicated that biofortified colored wheat varieties enhance lifespan of wild type flies in AL and DR conditions. However, the magnitude of increase was more under AL conditions than in DR, indicating that some DR-mediated effectors were responsible for the enhancement in lifespan in flies that were fed the colored wheat formulated AL diets. Since, the DR effectors were already active in the DR diet, a lesser increase in lifespan was observed under DR conditions. Moreover, the enhancement in lifespan did not show a direct correlation with the anthocyanin content of the wheat as flies that were fed blue wheat reproducibly lived longer than flies that were fed the black wheat formulated diet.

### 3.3 Effect of colored wheat flour DR diet on gene expression

DR induces changes in physiology and gene expression in diverse species including fruit flies (Plank et al., 2012; Pletcher et al., 2002; Rollins et al., 2019; Swindell, 2009; Whitaker et al., 2014; Wuttke et al., 2012). Hence, we examined the expression of genes that have been linked to DR-mediated lifespan extension in flies and mammals (Fig. 2). We predicted that if some of the beneficial effects of blue wheat formulated diet were mediated by conserved DR mediated pathways, then expression changes of genes regulating the relevant pathways would be observed in blue wheat AL diet and will not change further when flies were fed blue wheat DR diet. However, the expected expression changes would be observed for the mRNAs analyzed from flies that are shifted from CM AL to CM DR and WW AL to WW DR (Fig. 2A–L and Supplementary Fig. 1). Total RNA was extracted from wild type (*Canton S*) flies that were fed an AL or DR diet for 10 days and RT-PCR was performed to quantitate expression of DR-modulated and longevity genes including, *ampkalpha*, *srebp*, *catalase*, *sod2*, *PEPCK*, *cry*, *sima1/Hif1alpha*, *TORC*, *dilp6*, *relish*, *PI3K92E*, *atg8a*, *PRAS40*, *mitf*, *rpn11* and *nrf2*. Wild type flies that were fed a cornmeal or white wheat formulated DR diet for 10d expressed significantly higher levels of *ampkalpha* (CM DR vs AL: 2 fold; WW DR vs AL: 2.2 fold), *srebp* (CM DR vs AL: 2 fold; WW DR vs AL: 1.98 fold), *catalase* (CM DR vs AL: 1.9 fold; WW DR vs AL: 2.5 fold), *sod 2* (WW DR vs AL: 2.6 fold), *PEPCK* (WW DR vs AL: 2.6 fold), *Cry* (WW DR vs AL: 1.38 fold) as compared to wild types that were fed an AL diet (Fig. 2A–F). However, the expression of these genes did not change (*ampkalpha*, *srebp*, *sod2*, *PEPCK*, *Cry*, and *relish*) or decreased *(catalase)* upon shifting flies from BLW AL to BLW DR, indicating that the beneficial effects BLW formulated diet were mediated by these DR sensors and/or effectors (Fig. 2A–F). AMPK (AMP-activated protein kinase) is a key nutrient sensor that is activated in response to changes in the energy status of the cell. Activation of AMPK has been shown to extend lifespan in multiple species including *Drosophila* (Fontana et al., 2010; Greer et al., 2009; Kapahi et al., 2017). AMPK functions by promoting glycolysis and fatty acid oxidation in a short time frame and by increasing mitochondrial biogenesis, metabolism, and turnover. Moreover, tissue-specific upregulation of AMPK extends lifespan in *Drosophila* (Stenesen et al., 2013; Ulgherait et al., 2014). Sterol regulatory element binding proteins (SREBPs) are membrane bound transcription factors that function as master regulators of lipid metabolism and function by enhancing expression of enzymes involved in sterol and fatty acid biosynthesis (Seegmiller et al., 2002). Notably, CR-mediated lifespan extension is abolished in SREBP-1c knockout mice and in addition to its role as a regulator of lipid metabolism, SREBP also plays a role in CR-induced mitochondrial biogenesis and suppression of oxidative stress in adipose tissue (Fujii et al., 2017; Kobayashi et al., 2018). Phosphoenolpyruvate carboxy kinase (*PEPCK*) is a gluconeogenic enzyme whose activity increases upon CR in mice (Hagopian et al., 2003). *PEPCK* mRNA levels decrease with age in mammals and over-expression of the *C. elegans* homolog of PEPCK has been shown to increase increases health measures during ageing (Feng et al., 2016; Onken et al., 2020). PEPCK promotes autophagy, physical activity, defense against osmotic stress and oxidative stresses in different animal species (Frazier and Roth, 2009; Furukawa et al., 2015; Landis et al., 2012; Yuan et al., 2016; Yuan et al., 2012). Lastly, Cryptochromes (Cry) are critical components of the circadian clock that are modulated by DR and function as transcriptional repressors (Chaudhari et al., 2017; Patel et al., 2016). In contrast to the expression pattern of the above-described genes, flies that were fed a CM DR, WW DR or BLW DR expressed significantly higher levels of *sima/HIF1alpha*, *dilp6*, *PI3K92E*, nrf2, and *atg8a* (Fig. 2G–L) (Supplementary Fig. 1). Thus, indicating that the DR or longevity pathways pertaining to these gene expression changes were operating at similar levels in all the three diets. In summary, our gene expression analysis under AL and DR conditions indicates that the blue wheat formulated diet modulated expression of conserved genes involved in DR mediated enhancement of lifespan. These include DR regulators involved in antioxidant defense, gluconeogenesis, fat metabolism and circadian rhythm.

### 3.4 Effect of colored wheat formulated diet on paraquat-induced and hydrogen peroxide-induced oxidative stress in wild type flies

Paraquat (PQ) and hydrogen peroxide have been used to induce oxidative stress in diverse model organisms as both are generators of superoxide anion radicals and hydroxyl radicals, respectively (Bus and Gibson, 1984; Magwere et al., 2006; Phillips et al., 1989). In biological systems, redox cycling of PQ leads to the generation of superoxide anions, hydrogen peroxide and/or hydroxyl radicals that are highly reactive to tissue components. In addition, PQ also leads to depletion of cellular reducing equivalents that are essential for organismal function (Bus and Gibson, 1984). One strategy that has been used to increase survival rate upon PQ toxicity or hydrogen peroxide toxicity is long-term treatment with antioxidants and the effectiveness of exogenous anti-oxidative therapy has been demonstrated in cell and animal experiments (Hu et al., 2018; Magwere et al., 2006; Wang et al., 2018). We first verified that paraquat AL, paraquat DR or hydrogen peroxide diets were ingested to similar levels in the wheat- or cornmeal-supplemented diets by measuring food intake by EX-Q assay (Fig. 3A–C). Next, we determined whether the antioxidant activity of colored wheat flour formulated diet could rescue the paraquat sensitivity of wild type flies, by measuring survival of flies that were fed PQ supplemented AL or DR food containing different wheat flour varieties (Fig. 3D–G)(Tables 2A–2B). Adult wild type female flies that were fed biofortified colored wheat varieties were protected from the deleterious effects of the radicals generated by 15 mM PQ (Tables 2A–2B). In BLW AL diet with PQ, the median (Experiment 1: 6 days; Experiment 2: 4 days) and maximum lifespan (Experiment 1: 18 days; Experiment 2: 17 days) of wild type flies was significantly longer than the median (Experiment 1 and 2, 2 days) and maximum lifespan (Experiment 1: 7 days; Experiment 2, 8 days) of WW AL diet with PQ. Flies that were fed BW AL (Experiment 1: median, 6 days and maximum: 15 days; Experiment 2: median, 4 days and maximum, 15 days) or PW AL (Experiment 1: median, 3 days and maximum, 15 days; Experiment 2: median, 3 days and maximum, 18 days) diet with PQ also lived significantly longer than flies that were fed a WW AL diet with PQ. *Canton S* flies that were fed DR diets supple-mented with BLW or PW diet together with PQ lived significantly longer than flies that were fed a WW DR PQ diet (Fig. 3C–D) (Table 2B). These data indicated that colored wheat varieties improved tolerance of wild type flies to paraquat toxicity.

Resistance to oxidative stress can also be ascertained by survival analysis of flies in presence of hydrogen peroxide (Lee et al., 2017). To examine whether pre-consumption of biofortified wheat varieties strengthened the oxidative resistance of fruit flies in presence of hydrogen peroxide, wild type *Canton S* flies were fed the different wheat varieties during development and survival analysis of newly eclosed (2 days old) mated female flies was performed in presence of 5% hydrogen peroxide (Fig. 3E–F) (Table 2C). Adult *Canton S* flies that were reared on anthocyanin rich blue, purple, and black wheat formulated diets were protected from the harmful effects of hydrogen peroxide (Table 2C). The median lifespan of *Canton S* flies reared on blue wheat (Experiment 1: 90 h; Experiment 2: 96 h), purple wheat (Experiment 1: 90 h; Experiment 2: 96 h) and black wheat (Experiment 1: 84 h; Experiment 2: 96 h) was significantly higher than the median lifespan of *Canton S* flies on white wheat control (Experiment 1: 78 h; Experiment 2: 60 h). The magnitude of increase was the highest in flies that were fed the blue wheat diet (Experiment 1, WW vs BLW: χ^2^ = 31.59; Experiment 2: WW vs BLW: χ^2^ = 211.45) (Table 2C). Our analysis revealed that the *Canton S* flies that were fed the classical cornmeal diet also had a significantly higher tolerance to hydrogen peroxide induced stress (Experiment 1: *p*-value = 1.20E–07; Experiment 2: *p*-value = 0.00E+00). However, the flies that were fed a blue wheat formulated diet had a much longer maximum lifespan (Experiment 1: 138 h; Experiment 2: 144 h) than flies that were fed a cornmeal (Experiment 1: 126 h; Experiment 2: 132 h) or white wheat formulated diet (Table 2C). In summary, these data indicate that the biofortified wheat formulated diet enhanced resistance to oxidative stress and that the increased resistance does not correlate with the anthocyanin content as the diet formulated with black wheat provided a much lower protection than that conferred with prefeeding a blue wheat or purple wheat formulated diet.

### 3.5 Anthocyanin rich blue wheat flour increases resistance to paraquat-induced oxidative stress in SOD-deficient flies

To test the effectiveness of the blue colored wheat flour formulated diet in SOD-deficient flies, *sod2* knock down was driven ubiquitously in a steroid (RU-486) inducible manner with the Daughterless Geneswitch Gal4 *(daGS)* driver in adult flies (Fig. 4A). The crosses were set in cornmeal formulated diet and 2 days aged mated females were transferred to a food containing white or blue wheat flour in presence of RU-486 and paraquat (Fig. 4A–D). RT-PCR analysis of total RNA extracted from whole animals of *daGS/UAS sod2^RNAi^* female flies was performed to verify the knockdown of *sod 2* (Fig. 4B). Induction of the *UAS sod2^RNAi^* led to a 50.1 ± 7.3% decrease in *sod2* mRNA upon treatment with RU-486 for 5 days in female flies (Fig. 4B). Since, knockdown of Sod2 results in an increase in sensitivity to the toxic effects of paraquat, we measured survival of flies that were fed blue wheat flour or white wheat flour diet in presence of one-third the levels of Paraquat (5 mM) than what was used for the experiments with wild type flies (15 mM) (Fig. 3). *daGS/UAS sod2^RNAi^* female flies that were fed a blue wheat flour diet had a 45.8% increase in median lifespan in experimental trial 1 and 36.8% increase in median lifespan in the experimental trial 2 (Table 2D) (Fig. 4C–D).

The effect of ligand/RU-486 on the lifespan of the *DaGS* driver alone is reported previously and the effect of ligand on the UAS*sod2^RNAi^* line was examined and results indicated that the ligand does not influence lifespan of the transgenic lines (Bolukbasi et al., 2017) (Supplementary Fig. 2A–B). Since, our analysis has been done using the same genotype in different diets, the genetic background of the experimental strain in the different diets is identical and hence, the statistical difference in lifespan in the experiment can be attributed to the effect of blue wheat versus white wheat formulated diet. Taken together, these data indicate that anthocyanin containing blue wheat flour formulated diet enhanced the survival of *daGS/UAS Sod2^RNAi^* flies that were challenged with paraquat. Thus, indicating that blue wheat flour mediated protective effects in SOD-deficient flies even when it was fed in adult stages and the observed effects maybe mediated by the increased antioxidant properties of the blue wheat.

### 3.6 Anthocyanin containing blue wheat reverses the high fat diet induced lifespan phenotypes

Type 2 diabetes (T2D) and cardiovascular disease are two highly prevalent human metabolic diseases that are associated with intake of high fat diet (Szendroedi and Roden, 2009; van Herpen and Schrauwen-Hinderling, 2008). To test the effect of antioxidants in colored wheat for reversal of obesity induced phenotypes, lifespan was examined in wild type *Canton S* flies that were fed cornmeal or wheat flour supplemented HFD (Fig. 5A–G). Flies were maintained on cornmeal diet and 2–3 days old, mated males or females were sorted and transferred to a cornmeal high fat diet (CMHFD) for 7 days followed by transfer again to white wheat flour formulated HFD (WWHFD), blue wheat flour formulated HFD (BLWHFD) or cornmeal formulated HFD (CMHFD) for measurement of survivability (Fig. 4A). We first examined whether the CMHFD, WWHFD and BLWHFD were ingested to comparable levels by performing the EX-Q assay (Wu et al., 2020). No significant difference was seen in the food ingested by *Canton S* male or female flies that were fed a CMHFD, WWHFD or BLWHFD (Fig. 5B–C). Wild type (*Canton S*) male flies that were fed a blue wheat flour-supplemented HFD (blue dashed line) (BLWHFD) lived significantly longer than wild type flies that were fed a white wheat (WWHFD) (yellow line) flour-supplemented HFD or flies that were fed cornmeal HFD (CMHFD) (Fig. 5D–E) (Table 3). The median lifespan of male flies that were fed BLWHFD was 47% higher than flies that were fed a WWHFD and 42% higher than flies that were fed CMHFD in experiment 1 and 28% higher than flies that were fed WWHFD or CMHFD in experiment 2. In contrast, female *Canton S* flies that were fed either of the wheat formulated high fat diets (WWHFD or BWHFD) lived significantly longer than flies that were fed a CMHFD. The median lifespan of female flies that were fed a BLWHFD was 41.6% and 36.3% higher than female *Canton S* flies that were fed a CMHFD in experiment 1 and 2, respectively (Fig. 5F–G) (Table 3). The median lifespan of *Canton S* female flies that were fed a WWHFD diet was 41.6% longer than flies that were fed CMHFD in both experimental trials 1 and 2 (Fig. 5F–G) (Table 3). Thus, these results indicate that HFD results in sexually dimorphic lifespan phenotypes in biofortified wheat HFDs. Wild type male flies that were fed a blue wheat formulated high fat diet lived significantly longer than males that were fed a white wheat or cornmeal formulated high fat diet. In contrast, wild type female flies that were fed a white or blue wheat-formulated high fat diet lived significantly longer than flies that were fed a CMHFD.

### 3.7 Effect of wheat-formulated high fat diet on gene expression

To examine whether the sexually dimorphic effects of the blue colored wheat formulated high fat diet were due to differential expression of oxidative stress markers in males and females, we examined the gene expression pattern in flies that were fed high fat diet. We first tested whether the fatty acids in the coconut oil caused an increase in the lipid content by measuring the diameter of lipid droplets in the dissected fat tissue of adult flies by staining with Nile red. High fat diet fed flies exhibited larger lipid droplets in CM, WW and BLW formulated diets to the same extent as no statistical difference in lipid droplet size was observed between the three diets (Fig. 6A–C). To confirm that all three high fat diets were being ingested at comparable levels, EX-Q assay was performed (Fig. 6D). Next, we examined the expression of oxidative markers and antioxidant enzymes in flies that were fed a normal diet (CM, WW or BLW) or HFD (CM HFD, WW HFD, BLW HFD) (Fig. 6D–K). Quantitative RT-PCR was performed with total RNA extracted from whole flies that were fed cornmeal (CM), white wheat (WW) or blue wheat (BLW) normal diet (ND) or high fat diet (HFD). Feeding flies a diet supplemented with 25% coconut oil (HFD) for 5 days led to increase in oxidative stress markers such as *hsp70* (Fig. 6D and H). Heat shock proteins regulate stress resistance and lifespan in *Drosophila* (Ritossa, 1996). The heat shock proteins counteract the proteotoxic effects under stress conditions by functioning as chaperones during protein synthesis, assembly and degradation and the transcripts of *hsp70* are the first to increase upon oxidative stress. In contrast to male files, female flies expressed higher levels of *hsp70* in cornmeal, white wheat, or blue wheat normal diets. The magnitude of increase of *hsp70* upon HFD was significant in males that were fed wheat diets (~1.8 ± 0.2 fold in CM HFD diet relative to CM; 4.9 ± 0.3-fold in WW HFD versus WW; 12 ± 0.4-fold in BLW HFD versus BLW) than in females (1.4 ± 0.1-fold in CM HFD diet relative to CM; 1.4 ± 0.12-fold in WW HFD versus WW) and no increase in *hsp70* was see observed in female flies that were fed BLW HFD relative to BLW (Fig. 6H). Flies that were fed wheat formulated diets (White wheat or blue wheat) expressed significantly higher levels of *sod1* and *sod2* mRNAs in both males and females (Fig. 6E–F, 6I–J). The transcript levels of catalase were higher in male flies that were fed wheat diet but not in females (Fig. 6G–K). In summary, high fat diet led to a differential gene expression of oxidative stress markers and antioxidant enzymes in males and females. These differences in gene expression likely led to the differential survivability response of males and female flies that were fed blue colored and white wheat formulated high fat diet.

### 3.8 Effect of biofortified blue wheat on high fat induced on cardiac structural pathologies

High fat diet-induced obesity is a major risk factor for cardiomyopathy in humans and *Drosophila* (Birse et al., 2010; Szendroedi and Roden, 2009). Adult wild type flies that are fed a high fat diet (HFD) have increased levels of fat and myofibrillar disorganization within the cardiomyocytes and display locomotory defects, reduced lifespan and lipotoxicity (Birse et al., 2010; Borradaile and Schaffer, 2005; Diop and Bodmer, 2012; Liao et al., 2021; Schaffer, 2003; Wende and Abel, 2010). Despite fundamental anatomical differences between the *Drosophila* and mammals, several metabolic processes and key regulatory mechanisms operating in the heart are highly conserved, thus making fly an ideal model for unraveling the molecular mechanisms underlying cardiac diseases and for investigating the effect of diet on cardiac function and physiology (Diop and Bodmer, 2015). To determine whether biofortified blue wheat could alter the effects of HFD-induced structural defects in the heart, wild type *Canton S* flies were fed corn meal (CM), white wheat (WW) or blue wheat (BLW) diet throughout development and newly eclosed 2–3-day old flies were shifted to corresponding CMHFD, WWHFD or BLWHFD that contained 30% coconut oil. After 35 days in normal food (NF) or HFD diet, the abdomens were dissected to expose the heart tube and stained with phalloidin to examine the impact of the CMHFD, WWHFD or BLWHFD diet on the structure of the heart (Fig. 7A–B). The myofibrillar structure was severely disorganized in the heart of WWHFD fed flies as compared to BLWHFD and CMHFD fed flies (Fig. 7A) indicating that feeding flies a blue wheat formulated diet was able to delay the HFD-induced defects in the heart structure. The heart tube diameter constriction has been reported as a phenotype associated with the consumption of HFD in fruit flies (Birse et al., 2010). To determine whether the heart tubes in WWHFD and CMHFD fed flies were more constricted than the heart tube in BLWHFD fed flies, the width of the heart tubes was measured in the abdominal segments 2 and 3 (A2 and A3) and the A2-A3 junction. Compared to CM or CMHFD fed *Canton S* flies, the heart tube of *Canton S* flies that were fed white wheat normal food or HFD formulated (WW) diets were wider in the abdominal segment 3 (A3) (Fig. 7A and E). The width of the heart tube did not vary significantly between the normal food and HFD fed flies (Fig. 7C–E). Taken together, these data suggest that the biofortified blue wheat formulated diet moderately prevents the adverse effects of HFD on the heart.

## 4 Discussion

### 4.1 Evidence for antioxidants in healthy ageing

Antioxidants reduce the rate of cell death by neutralizing reactive oxygen species that cause oxidative cellular damage during organismal ageing. Oxidative damage is a hallmark of ageing and is characterized by an impaired antioxidant defense together with an increase in the generation of reactive oxygen species (ROS) (Kirkwood, 2005; Sohal and Weindruch, 1996). Here, we have examined the effectiveness of anthocyanin enriched wheat formulated diets in prolonging lifespan under different dietary contexts. Amongst the different colored wheat varieties tested, blue wheat formulated diet enhanced lifespan under AL, DR, and high fat diet. In addition, blue wheat formulated diet also provided significant protection against oxidative stress induced by paraquat and hydrogen peroxide. Our gene expression analysis under AL and DR conditions indicates the involvement of DR-effectors such as AMPK alpha, SREBP and Cry in blue wheat mediated lifespan extension (Fig. 2). Our data also indicate that biofortified wheat varieties show differential effects in enhancing lifespan and that the content of anthocyanins does not correlate with the beneficial effects. Despite having been previously reported to have the highest anthocyanin content, black wheat formulated diet did not increase longevity compared to the control white wheat diet and blue wheat consistently enhanced lifespan in different dietary contexts (Fig. 1C–D) (Table 1A) (Garg et al., 2016). These data lend support to the already existing evidence that more amount of antioxidants of one kind (500 mg vitamin C tablet as opposed to <100 mg in a cup of fresh fruit) are not always beneficial and that differences in the amount and type of antioxidant in supplements versus natural foods (e.g. polyphenols, flavonoids and proanthocyanins) might influence health outcomes (Hennekens et al., 1996; Klein et al., 2011). In addition, there are studies that indicate that combinations of antioxidants are protective against development of age-related macular degeneration (Age-Related Eye Disease Study Research, 2001).

In summary our data indicates that intake of antioxidant rich biofortified cereal diets enhances lifespan and healthspan in *Drosophila* and further emphasizes the utility of testing the health promoting effects of dietary interventions in diverse dietary contexts that are more representative of the diverse food consumption patterns (Bhupathiraju et al., 2013; Joshipura et al., 2001; Miller et al., 2017).

### 4.2 Biofortification of food crops as an alternative strategy for promoting healthy ageing

Micronutrient deficiencies affect about a third of the world population and biofortification of food crops has been utilized as a cost-effective and feasible strategy to increase the nutritional value of food and improve the health of populations that have limited access to diverse diets and other micronutrient interventions (Bouis and Saltz-man, 2017). Wheat is one of the most widely grown staple crop in which conventional breeding approaches have been utilized for improvement in the content of iron, zinc, provitamin A, beta-carotene and anthocyanin content (Garg et al., 2018). Here, we have evaluated the efficacy of biofortified wheat flour formulated diet in modulating lifespan in a short-lived invertebrate model. This is the first study to examine the long-term health impact of consumption of native biofortified anthocyanin-enriched wheat varieties developed by conventional plant -breeding on *Drosophila* ageing. A recent study by Sharma et al., 2020 examined the short-term effect of some of these wheat varieties (black, purple and white wheat) in an HFD induced mouse model and showed that biofortified black wheat conferred health benefits by modulating expression of genes involved in antioxidative response and fatty acid beta oxidation response (Sharma et al., 2020). Our expression analysis under *ad libitum* and dietary restriction conditions indicates that blue wheat diet modulates expression of genes involved in different DR-mediated pathways including nutrient signaling, gluconeogenesis, mitochondrial biogenesis, antioxidative response and circadian rhythm. Several studies in the fruit fly model have reported beneficial effects of anthocyanin fruits such as berries, colored grapes, sweet cherries and colored fruits and vegetables (Yi et al., 2021). However, these fruits are largely inaccessible to the majority of the populations in underdeveloped and developing countries. Moreover, the abundance of sugar in the anthocyanin rich fruits limits the health benefits of these anthocyanin rich fruits in obese populations. Taken together our findings have important implications for development of biofortified crops for enhancing healthy lifespan of the rapidly growing elderly populations worldwide and future strategies aimed at development and evaluation of combinations of natural plant-based crops will likely aid in the design of an optimal diet for prevention and/or delay of age-related chronic diseases and promoting healthy lifespan.

## Supplementary Material

Supplementary Material

## Figures and Tables

**Table 1A:** Lifespan analysis of *Canton S* flies in wheat or cornmeal formulated diets.

Strain/diet	Lifespan (days)		*p*-Value^*^	χ^2^^*^	*p*-Value^#^
	Maximum (number of flies)	Median			
Experiment 1					
White wheat (WW)	60 (159)	38	0.4511	0.57	1
Black wheat (BW)	60 (140)	36			
White wheat (WW)	60 (159)	38	3.3E−06	21.62	3.00E−05
Blue wheat (BLW)	68 (160)	42			
White wheat (WW)	60 (159)	38	0.5181	0.42	1
Purple wheat (PW)	64 (160)	36			
White wheat (WW)	60 (159)	38	0.2523	1.31	1
Cornmeal (CM)	62 (160)	40			
Experiment 2					
White wheat (WW)	60 (140)	38	0.3486	0.88	1
Black wheat (BW)	62 (140)	34			
White wheat (WW)	60 (140)	38	4.10E− 05	16.84	0.0004
Blue wheat (BLW)	66 (100)	40			
White wheat (WW)	60 (140)	38	0.0435	4.07	0.3918
Purple wheat (PW)	66 (180)	36			
White wheat (WW)	60 (140)	38	0.0958	2.77	0.8621
Cornmeal (CM)	60 (160)	38			

* For statistical comparison of survival curves, *p*-values and χ^2^ were calculated with log rank test.

# *p*-Value calculated after applying Bonferroni correction.

**Table 1B:** Lifespan analysis of *Canton S* flies in wheat-based *Ad libitum* (AL) diets.

Strain/diet	Lifespan (days)		*p*-Value^*^	χ^2^^*^	*p*-Value^#^
	Maximum (number of flies)	Median			
Experiment 1					
White wheat AL	42 (124)	22	0.1122	2.52	1
Cornmeal AL	42 (99)	28			
White wheat AL	42 (124)	22	0.0005	12.09	0.0046
Purple wheat AL	54 (136)	28			
White wheat AL	42 (124)	22	7.40E−06	20.1	0.0001
Black wheat AL	50 (158)	28			
White wheat AL	42 (124)	22	3.80E−07	25.81	3.40E− 06
Blue wheat AL	64 (158)	30			
Experiment 2					
White wheat AL	42 (115)	24	0.8879	0.02	1
Cornmeal AL	40 (100)	24			
White wheat AL	42 (115)	24	3.80E-05	16.98	0.0003
Purple wheat AL	54 (160)	28			
White wheat AL	42 (115)	24	1.20E−06	23.64	1.00E−05
Black wheat AL	54 (126)	30			
White wheat AL	42 (115)	24	0.00E+00	44.14	0.00E+00
Blue wheat AL	64 (160)	32			

* For statistical comparison of survival curves, *p*-values and χ^2^ were calculated with log rank test.

# *p*-Value calculated after applying Bonferroni correction.

**Table 1C:** Lifespan analysis of *Canton S* flies in wheat-based DR diets.

Strain/diet	Lifespan (days)		*p*-Value^*^	χ^2^^*^	*p*-Value^#^
	Maximum (number of flies)	Median			
Experiment 1					
White wheat DR	62 (139)	38	3.70E− 05	17	0.0003
Cornmeal DR	68 (120)	42			
White wheat DR	62 (139)	38	0.0064	7.44	0.0574
Purple wheat DR	74 (136)	42			
White wheat DR	62 (139)	38	0.0442	4.05	0.3978
Black wheat DR	66 (140)	38			
White wheat DR	62 (139)	38	0.00E+00	45.87	0.00E+00
Blue wheat DR	72 (175)	48			
Experiment 2					
White wheat DR	62 (138)	36	0.0001	14.67	0.0012
Cornmeal DR	70 (119)	42			
White wheat DR	62 (138)	36	0.0172	5.68	0.1549
Purple wheat DR	74 (160)	40			
White wheat DR	62 (138)	36	0.0002	13.47	0.0022
Black wheat DR	64 (126)	40			
White wheat DR	62 (138)	36	0.00E+00	40.06	0.00E+00
Blue wheat DR	74 (136)	46			

DR, Dietary restriction.

* For statistical comparison of survival curves, *p*-values and χ^2^ were calculated with log rank test.

# *p*-Value calculated after applying Bonferroni correction.

**Table 2A:** Lifespan analysis of *Canton S* flies in wheat-based *ad libitum* (AL) paraquat diets.

Diet	Lifespan (days)		*p*-Value^*^	χ^2^^*^	*p*-Value^#^
	Maximum (number of flies)	Median			
Paraquat AL Experiment 1					
White wheat AL (WW AL PQ)	7 (99)	2	0.00E+00	66.54	0.00E+00
Black wheat AL (BW AL PQ)	15 (79)	6			
White wheat AL (WW AL PQ)	7 (99)	2	0.00E+00	82.35	0.00E+00
Blue wheat AL (BLW AL PQ)	18 (99)	6			
White wheat AL (WW AL PQ)	7 (99)	2	1.50E−07	27.54	6.20E− 07
Purple wheat AL (PW AL PQ)	15 (86)	3			
White wheat AL (WW AL PQ)	7 (99)	2	0.00E+00	43.17	0.00E+00
Cornmeal AL (CM AL PQ)	13 (71)	4			
Paraquat AL Experiment 2					
White wheat AL (WW AL PQ)	8 (84)	2	9.70E−08	28.42	3.90E− 07
Black wheat AL (BW AL PQ)	15 (78)	4			
White wheat AL (WW AL PQ)	8 (84)	2	0.00E+00	55.63	0.00E+00
Blue wheat AL (BLW AL PQ)	17 (74)	4			
White wheat AL (WW AL PQ)	8 (84)	2	1.50E−08	32.08	6.00E− 08
Purple wheat AL (PW AL PQ)	18 (77)	3			
White wheat AL (WW AL PQ)	8 (84)	2	0.00E+00	61.28	0.00E+00
Cornmeal AL (CM AL PQ)	14 (78)	7			

AL, *Ad libitum;* PQ, Paraquat.

* For statistical comparison of survival curves, *p*-values and χ^2^ were calculated with log rank test.

# *p*-Value calculated after applying Bonferroni correction.

**Table 2B:** Lifespan analysis of *Canton S* flies in wheat-based DR paraquat diets.

Diet	Lifespan (days)		*p*-Value^*^	χ^2^^*^	*p*-Value^#^
	Maximum (number of flies)	Median			
Paraquat DR Experiment 1					
White wheat DR (WW DR PQ)	10 (134)	2	2.90E− 09	35.29	1.2E− 08
Black wheat DR (BW DR PQ)	11 (95)	4			
White wheat DR (WW DR PQ)	10 (134)	2	0.00E+00	86.52	0.00E+00
Blue wheat DR(BLW DR PQ)	12 (101)	6			
White wheat DR (WW DPQ)	10 (134)	2	0.00E+00	48.88	0.00E+00
Purple wheat DR (PW DPQ)	16 (78)	5			
White wheat DR (WW DPQ)	10 (134)	2	2.20E− 08	31.28	9.00E− 08
Cornmeal DR (CM DR PQ)	12 (88)	3			
Paraquat DR Experiment 2					
White wheat DR (WW DR)	10 (125)	3	6.80E− 06	20.24	2.70E− 05
Black wheat DR (BW DR)	14 (76)	4			
White wheat DR (WW DR)	10 (125)	3	0.00E+00	67.01	0.00E+00
Blue wheat DR (BLW DR)	15 (80)	6			
White wheat DR (WW DR)	10 (125)	3	0.00E+00	40.15	0.00E+00
Purple wheat DR (PW DR)	17 (75)	5			
White wheat DR (WW DR)	10 (125)	3	2.40E− 05	17.85	0.0001
Cornmeal DR (CM DR)	12 (66)	4			

DR, dietary restriction; PQ, Paraquat.

* For statistical comparison of survival curves, p-values and χ^2^ were calculated with log rank test.

# *p*-Value calculated after applying Bonferroni correction.

**Table 2C:** Survival analysis of *Canton S* flie*s* in presence of 5% hydrogen peroxide.

Diet	Lifespan (hours)		*p*-Value^*^	χ^2^^*^	*p*-Value^#^
	Maximum (number of flies)	Median			
Experiment 1					
White wheat (WW)	114 (100)	78	3.10E−08	30.67	1.20E−07
Cornmeal (CM)	126 (102)	90			
White wheat (WW)	114 (100)	78	1.40E−07	27.79	5.40E− 07
Purple wheat (PW)	132 (106)	90			
White wheat (WW)	114 (100)	78	0.0078	7.08	0.0311
Black wheat (BW)	144 (105)	84			
White wheat (WW)	114 (100)	78	1.90E−08	31.59	7.70E− 08
Blue wheat (BLW)	138 (104)	90			
Experiment 2					
White wheat (WW)	84 (118)	60	0.00E+00	146.4	0.00E+00
Cornmeal (CM)	132 (142)	84			
White wheat (WW)	84 (118)	60	0.00E+00	197.57	0.00E+00
Purple wheat (PW)	132 (114)	96			
White wheat (WW)	84 (118)	60	0.00E+00	175.45	0.00E+00
Black wheat (BW)	144 (129)	96			
White wheat (WW)	84 (118)	60	0.00E+00	211.45	0.00E+00
Blue wheat (BLW)	144 (105)	96			

* For statistical comparison of survival curves, p-values and χ^2^ were calculated with log rank test.

# p-Value calculated after applying Bonferroni correction.

**Table 2D:** Lifespan analysis of *UAS SOD*^*RNAi*^ flies in wheat-based paraquat diets.

Genotype/diet	Lifespan (hours)		*p*-Value^*^	χ^2^^*^
	Maximum (number of flies)	Median		
Experimental trial 1				
*daGS > UAS SOD ^RNAi^* WW	792 (96)	156	0.0240	5.096
*daGS > UAS SOD ^RNAi^* BLW	876 (106)	288		
Experimental trial 2				
*daGS > UAS SOD ^RNAi^* WW	828 (120)	288	2.50E− 07	26.6
*daGS > UAS SOD ^RNAi^* BLW	876 (106)	456		

WW, white wheat flour diet; BLW, blue wheat flour diet.

* For statistical comparison of survival curves, *p*-values and χ^2^ was calculated with log rank test.

**Table 3:** Lifespan analysis of *Canton S* male and female flies on wheat based high fat diet.

Strain/ diet	Lifespan (days)		*p*-Value^*^	χ^2^^*^	*p*-Value^#^
	Maximum (number of flies)	Median			
Experiment 1 (males)					
WWHFD	46 (103)	20	0.00E+00	56.99	0.00E+00
BLWHFD	62 (106)	38			
WWHFD	46 (103)	20	0.0012	10.54	0.0023
CMHFD	52 (100)	22			
BLWHFD	62 (106)	38	1.30E−05	19.02	2.60E− 05
CMHFD	52 (100)	22			
Experiment 2 (males)					
WWHFD	38 (113)	20	5.40E−09	34.04	1.10E−08
BLWHFD	56 (108)	28			
WWHFD	38 (113)	20	0.0367	4.36	0.0735
CMHFD	46 (81)	20			
BLWHFD	56 (108)	28	0.0003	13.02	0.0006
CMHFD	46 (81)	20			
Experiment 1 (females)					
WWHFD	54 (145)	24	0.6745	0.1763	1
BLWHFD	56 (137)	24			
WWHFD	54 (145)	24	1.80E−05	18.37	0.0001
CMHFD	46 (118)	14			
BLWHFD	56 (137)	24	0.0002	14.11	0.0009
CMHFD	46 (118)	14			
Experiment 2 (females)					
WWHFD	50 (165)	24	0.1037	2.65	0.5187
BLWHFD	56 (126)	22			
WWHFD	50 (165)	24	0.0263	4.94	0.1313
CMHFD	46 (109)	14			
BLWHFD	56 (126)	22	0.0013	10.37	0.0064
CMHFD	46 (109)	14			

WWHFD, white wheat flour high fat diet; BLWHFD, blue wheat flour high fat diet; CMHFD, cornmeal high fat diet.

* For statistical comparison of survival curves, *p*-values and χ^2^ were calculated with log rank test.

# *p*-Value calculated after applying Bonferroni correction.
