# Supplementary material for: Evaluation of lifespan promoting effects of biofortified wheat in Drosophila melanogaster
Source: Exp Gerontol. Author manuscript; Available in PMC 2022 Jul 9. (PMC7613042; doi:10.1016/j.exger.2022.111697)
Supplement: Supplementary Material [file EMS147807-supplement-Supplementary_Material.docx]

*
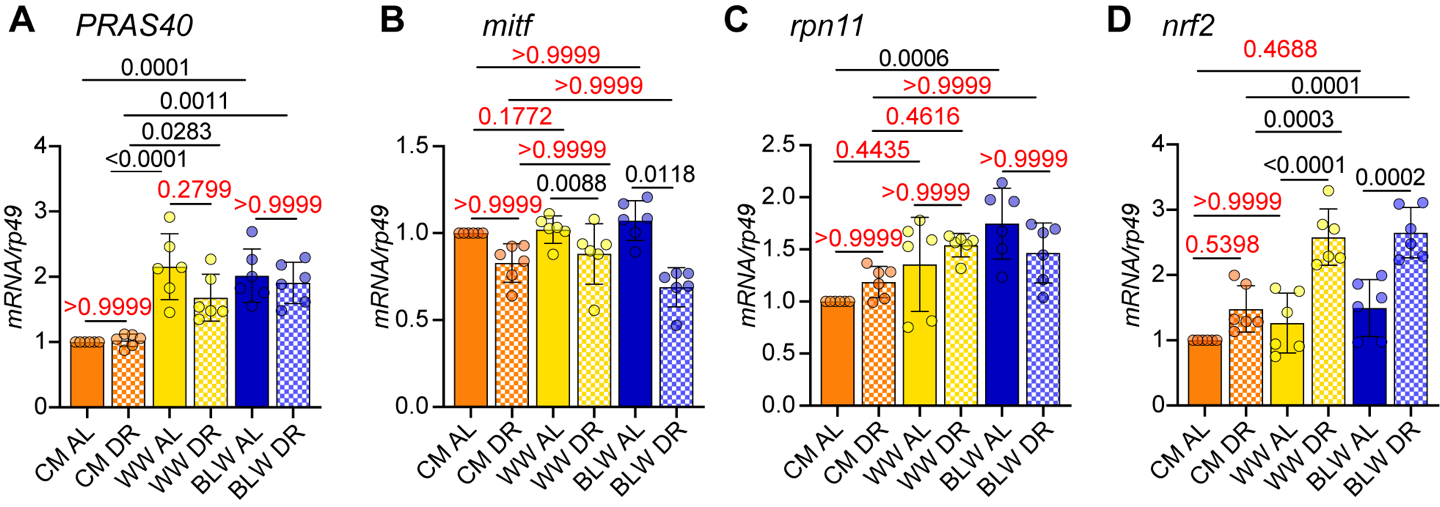
*

**Supplementary Figure 1.** **Effect of wheat formulated diet on the expression of age and DR-modulated genes.**  **(A-D)** Quantitative RT-PCR analysis of *PRAS40, mitf, rpn11,* and *nrf2* from RNA extracted from *Canton S* female flies that were fed Cornmeal *Ad libitum* (CM AL) (orange bar), cornmeal DR diet (CM DR) (orange pattern bar), white wheat AL (WW AL)(yellow bar), white wheat DR (WW DR)(yellow pattern bar), blue wheat AL (BLW AL)( blue bar) and blue wheat DR (BLW DR) (blue pattern bar) for 10 days. (A) Expression analysis of *PRAS40* (P-value calculated by ordinary one-way ANOVA 4.93E-07). **(B)** Expression analysis of *mitf* (P-value calculated by ordinary one-way ANOVA 6.91E-05. **(C)** Expression analysis of *rpn11* (P-value calculated by ordinary one-way ANOVA 7.59E-04).  **(D)** Expression analysis of *nrf2* (P-value calculated by ordinary one-way ANOVA 7.68E-09). Expression levels were normalized to *rp49*. Data are represented as mean ± SD, n=3. Adjusted P-values after applying Bonferroni’s adjusted p-values are represented in the figure and we used an α level of 0.05 to assess statistical significance.

Genotypes of strains used in this figure: **(A-D):** *Canton S*.

**
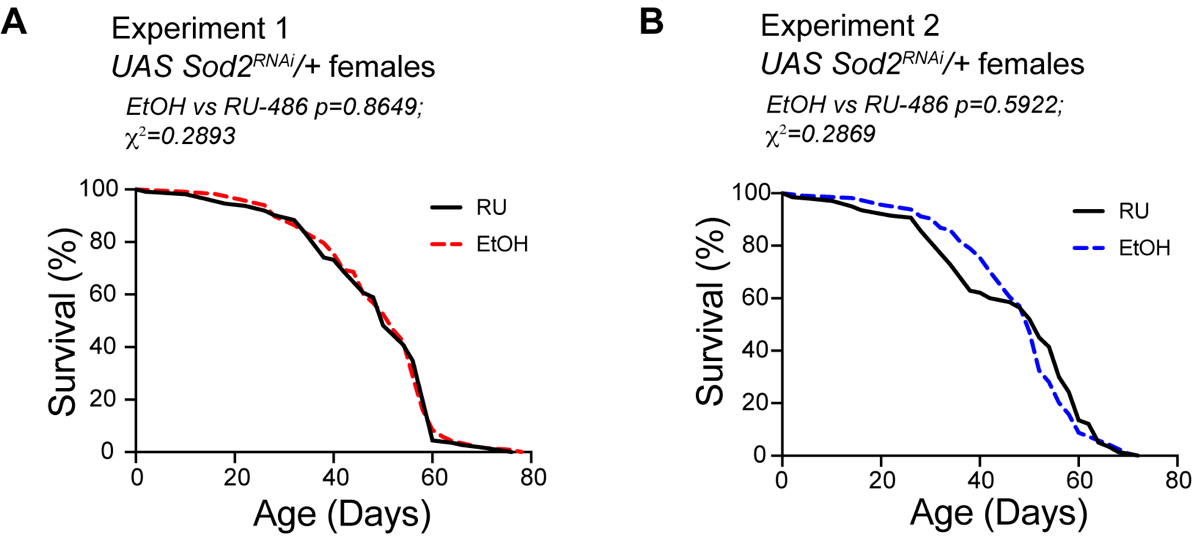
**

**Supplementary Figure 2. Effect of RU-486 on lifespan of *UAS Sod2^RNAi^ X w^1118^* flies.** (A-B) Crosses were established between *UAS Sod2^RNAi^* and *w^1118^* and the female progeny was sorted into two groups (Ethanol and RU-486). No significant difference was seen in the survivability in presence or absence of RU-486. The number of flies in Experiment 1: Ethanol, n=118 and RU-486, n=112; and in Experiment 2: Ethanol, n=114 and RU-486, n=140. For statistical comparison of survival curves, p-values and χ^2^ were calculated with log rank test are indicated in the figure panels.

Genotype of strain used in the figure: *w^1118^*; *UAS Sod2^RNAi^/+, +/+;P{y[+t7.7] v[+t1.8]=TRiP.GL01015}attP40/+*.
